# Recent Developments in Microfluidic Technologies for Central Nervous System Targeted Studies

**DOI:** 10.3390/pharmaceutics12060542

**Published:** 2020-06-11

**Authors:** Maria Inês Teixeira, Maria Helena Amaral, Paulo C. Costa, Carla M. Lopes, Dimitrios A. Lamprou

**Affiliations:** 1 UCIBIO-REQUIMTE, MedTech - Laboratory of Pharmaceutical Technology, Department of Drug Sciences, Faculty of Pharmacy, University of Porto, Rua de Jorge Viterbo Ferreira, 228, 4050-313 Porto, Portugal; up201410537@ff.up.pt (M.I.T.); hamaral@ff.up.pt (M.H.A.); pccosta@ff.up.pt (P.C.C.); 2 School of Pharmacy, Queen’s University Belfast, 97 Lisburn Road, Belfast BT9 7BL, UK; 3 FP-ENAS/CEBIMED, Fernando Pessoa Energy, Environment and Health Research Unit/Biomedical Research Centre, Faculty of Health Sciences, Fernando Pessoa University, Rua Carlos da Maia, 296, 4200-150 Porto, Portugal

**Keywords:** Neurodegenerative diseases (NDs), blood-brain barrier (BBB), central nervous system (CNS), microfluidics, organ-on-a-chip, brain-on-a-chip, brain delivery, drug delivery, nanoparticles (NPs), nanocarriers

## Abstract

Neurodegenerative diseases (NDs) bear a lot of weight in public health. By studying the properties of the blood-brain barrier (BBB) and its fundamental interactions with the central nervous system (CNS), it is possible to improve the understanding of the pathological mechanisms behind these disorders and create new and better strategies to improve bioavailability and therapeutic efficiency, such as nanocarriers. Microfluidics is an intersectional field with many applications. Microfluidic systems can be an invaluable tool to accurately simulate the BBB microenvironment, as well as develop, in a reproducible manner, drug delivery systems with well-defined physicochemical characteristics. This review provides an overview of the most recent advances on microfluidic devices for CNS-targeted studies. Firstly, the importance of the BBB will be addressed, and different experimental BBB models will be briefly discussed. Subsequently, microfluidic-integrated BBB models (BBB/brain-on-a-chip) are introduced and the state of the art reviewed, with special emphasis on their use to study NDs. Additionally, the microfluidic preparation of nanocarriers and other compounds for CNS delivery has been covered. The last section focuses on current challenges and future perspectives of microfluidic experimentation.

## 1. Introduction

Neurodegenerative diseases (NDs) such as Parkinson’s disease (PD), Alzheimer’s disease (AD), Amyotrophic lateral sclerosis (ALS), Multiple sclerosis (MS) or Huntington’s disease (HD) are among the disorders with the highest incidence worldwide, with their increase alarming when it comes to the elderly population [1,2]. In light of their prevalence, they represent a critical public health and economic concern [3]. These ailments are marked by different clinical features, depending on the involved regions of the central nervous system (CNS), with some causing cognitive and memory dysfunction, and others instigating motor impairment [4]. Several biological mechanisms contribute to the appearance and chronic progression of NDs, including oxidative stress, mitochondrial dysfunction, neural excitotoxicity, protein aggregation, depletion or deficient synthesis of neurotransmitters or their degradation in the synaptic cleft due to higher enzymatic activity, and gross malfunction of the blood-brain barrier (BBB) [5].

The BBB is a highly complex dynamic interface between the bloodstream and the brain, effectively protecting the CNS from potentially harmful external elements and maintaining cerebral homeostasis [6,7]. BBB disruption, linked with increased permeability, impairment of influx/efflux mechanisms and infiltration of toxins and immune cells in the CNS, is a common factor to many NDs [7]. The establishment of in vitro biomimetic platforms that can precisely replicate the functional/structural properties of the BBB and enable the monitoring of changes to its integrity, could therefore enhance the understanding of NDs, as well as accelerate the research and development of innovative therapies [6,7]. Ideally, to faithfully recapitulate the human brain endothelium, in vitro BBB models should possess some of its essential features, including interactions between different types of cells (e.g., vascular endothelial cells, astrocytes and pericytes), a 3D vessel-like structure of the endothelial cells, the presence of a basement membrane and flow-induced sheer stress [6,7].

Microfluidics is an intersectional field that deals with systems that process or manipulate nanolitre amounts of fluids, using channels ranging in size from tens to hundreds of micrometres [8]. Advances in microfluidic technology have led to the generation of devices (so-called organs-on-a-chip) that can be standardized at various scales and flow regimes, allowing the simulation of BBB in vivo physiology through reconstitution of its multicellular architecture and physicochemical microenvironment [7,9,10]. Conventional 2D or 3D systems cannot produce such levels of tissue and organ functionality [10]. By providing a highly controlled cellular microenvironment, organs-on-a-chip are suitable tools to assess the responses of the BBB to physiological and pathological stimuli, including analysis of metabolic, genetic and biochemical activities [7,10]. Furthermore, they also allow for real-time, high-resolution imaging [10]. Thus, these types of models have huge potential for a wide variety of applications, including the study of NDs aetiology, biomarker identification, neurotoxicity testing or high-throughput screening of CNS drug candidates [7,10].

Currently, none of the available medicines are able to stop or slow the continuous loss of neurons that occurs in NDs, with therapy being majorly focused on symptomatic relief [4]. Considering that the number of cases is expected to rise, with the World Health Organization (WHO) predicting that in 20 years’ time NDs will become the second leading cause of death, there is an urgent and unmet need to come up with more effective therapies [11,12]. However, the treatment of brain diseases is severely hindered by the BBB [13]. Under normal circumstances, the BBB only allows selective passage of molecules essential to the functioning of the brain, restricting the entrance of 98%, or more, of therapeutics [14].

In this context, nanotechnology-based approaches such as nanoparticles (NPs) present an opportunity to rethink and retackle the adversities connected to poor BBB permeability when delivering drug candidates/diagnostic agents to the CNS. These targeting strategies have the potential to make use of receptors and transporters commonly expressed at the luminal side of the brain endothelium to transport therapeutics across the BBB, leveraging internalization mechanisms that differ from the pathways taken by the majority of free drugs [15,16]. NPs can have significant advantages over traditional pharmaceutical formulations, including biocompatibility and biodegradability of their materials, ease of scaling-up, reduced toxicity, controlled and targeted delivery, enhanced bioavailability and dissolution rate, improved pharmacokinetics and therapeutic effects, and protection of encapsulated biomolecules [17,18].

Conventional methods of NPs preparation are sometimes associated with lack of precise control and high batch-to-batch variations. In this area, microfluidics has emerged as a forthcoming and noteworthy alternative, enabling fine tuning of process parameters and optimization. Microfluidic platforms allow for better control and reproducibility over NPs properties, including size distribution, zeta potential or ligand density, which are critical for their clinical translation as prime drug carriers [8,19,20]. Importantly, the volume of the fluids used, enables a substantial reduction in the consumption of reagents, being cost-effective [8]. Furthermore, given that the process of formation of NPs occurs in a continuous flow pattern, with preservation of controlled hydrodynamic conditions inside the microchannels, it is possible to obtain highly monodisperse nanosystems, avoiding batch-to-batch variability [8,19].

This review provides an overview of the most recent advances in microfluidic devices for CNS-targeted studies. Firstly, the importance of the BBB will be addressed, and different experimental BBB models will be briefly discussed. Subsequently, microfluidic-integrated BBB models (BBB/brain-on-a-chip) are introduced and the state of the art reviewed, with special emphasis on their use to study NDs. Additionally, the microfluidic preparation of nanocarriers and other compounds for CNS delivery have been covered. The last section focuses on current challenges and future perspectives of microfluidic experimentation.

## 2. The Blood-Brain Barrier (BBB)

**Figure 1:**
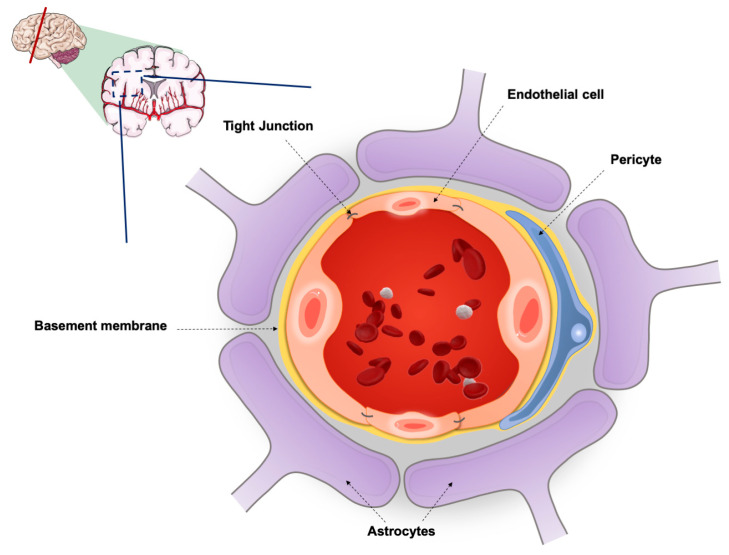
Schematic representation of a neurovascular unit (NVU) cross section in the blood-brain barrier (BBB).

The BBB is a highly specialized capillary wall, primarily comprised of brain endothelial cells and its basement membrane. Adherens and tight junctions (TJs) form cell–cell adhesions that maintain the integrity of the microvasculature but severely limit the paracellular transport, thus making transcellular diffusion the main transport mechanism of molecules between the blood and the brain, and vice-versa [21]. Brain capillaries are encircled by astrocytic perivascular endfeet, which contribute to the regulation of ion concentration and clearance of neurotransmitters, and by pericytes through the basal laminal, having a crucial role in the regulation of blood flow, BBB integrity and transport. Neurons and microglia also participate in the formation of the BBB, establishing with the astrocytes and pericytes a dynamic functional unit, called neurovascular unit (NVU) (Figure 1). Functional signalling and interaction between these components is the basis for BBB stability and its response to pathophysiological stimuli [21,22].

As a physical and biochemical barrier, the BBB has an essential role in brain homeostasis, limiting the entry of harmful toxins and pathogens present in the blood, while supplying the fundamental nutrients and molecules needed for normal cell function [22]. Although the restrictive nature of the BBB is extremely important for the protection of the CNS, it poses a challenge when it comes to the management of brain diseases, since therapeutic molecules are not able to penetrate the basement membrane and reach the intended target site. The entry of therapeutic candidates into the brain parenchyma depends on several factors [21]. As previously mentioned, paracellular transport is very limited and negligible. Low molecular weight (less than 400–500 Da) lipophilic substances, preferably neutrally charged, permeate the brain through passive transcellular diffusion [23]. It is also possible to take advantage of some active transport mechanisms in order to assist drug penetration across the BBB, including [22,23]:

- Carrier-mediated transcytosis (binding of a molecule to a protein carrier on the apical side of the capillary wall, followed by its conformational change and transport of said molecule to the other side of the membrane).
- Receptor-mediated transcytosis (binding of a particular ligand to a specific receptor, such as transferrin or low-density lipoprotein receptors, with the formation of a ligand-receptor complex which is carried through the cytoplasm and released on the basolateral side of the BBB).
- Adsorptive transcytosis (electrostatic interaction between positively charged molecules and the negatively charged plasma membrane).

Despite the fact that therapeutic molecules might get access to the CNS by passive or active transport, many are subsequently removed by efflux pumps such as the P-glycoprotein (P-gp) or isoforms of the multidrug resistance protein (MRP), resulting in the extrusion of the drugs back into the blood circulation and further complicating the effective treatment of brain disorders [22,24]. Hence, with the aim to optimize the therapeutic effect of the drugs, some tactics focus on evading or inhibiting the efflux pumps [23]. An interesting aspect to consider is that, depending on the CNS disorder, distinct expression profiles of efflux transporters are observed. For instance, in MS, there is a decrease in endothelial cell P-gp, whereas MRP1 or MRP2 remain unchanged [25]. In turn, in ALS, there is an increase in the P-gp but not MRP 1, 2, 4 or 5 expression in the cerebral cortex and spinal cord [26]. This suggests that a rather complex interplay between age-related decrease and disease-related stimulation, may lead to differences in the levels of such efflux pumps [27].

There is a recognized link concerning pathological impairment/disruption of the BBB and the development of NDs. Several morphological alterations of cellular elements, as well as changes at a molecular level, lead to BBB breakdown, with ensuing neural inflammation, dysfunction and degeneration. Numerous factors may be involved in the BBB disruption, including reactive oxygen species (ROS), angiogenic factors, inflammatory cytokines, mitochondrial dysfunction, aberrant intracellular signalling, autoantibodies, leukocyte adhesion and pathogens [28]. Degenerative changes in the microvasculature persist as the disease evolves, and may be reflected in reduced expression of TJs or adherens junctions, defective expression or function of transporters, upregulation of luminal adhesion molecules, astrocyte loss, pericyte detachment, insufficient clearance function or disrupted basement membrane [28,29,30]. The implications vary from unregulated molecular and ionic fluxes, extravasation of plasma proteins, entry of pathogens and toxins, to the onset of a central inflammatory response with microglial and astroglial activation, as well as release of cytokines and chemokines [28,31]. However, it can be said that the single most important outcome is that the integrity of the BBB is severely compromised, mainly manifesting as increased barrier permeability [28].

In recent years, exponential focus has been given to alterations of the extracellular matrix (ECM) of the basement membrane as a key contributor to the pathological process of NDs. For instance, it has been shown that matrix metalloproteinases (MMPs), in cases of chronic neurodegeneration, drive forth the progression of symptomatology in AD and PD [32]. Increasing evidence also indicates that dysbiosis of gut microbiome may influence and contribute to the onset and progression of a number of diseases, including NDs [33].

Overall, it is critical to have a better understanding and translation of the BBB physiological and pathological processes that precede and increase vulnerability to NDs, since it might offer new avenues to help identify novel strategies and therapeutic targets for drug discovery and development.

## 3. Experimental BBB Models

### 3.1. In Vivo Models

BBB research is met with challenging difficulties given its complexities and relative inaccessibility. Reconstituting critical features of the BBB, as well as of NDs, is imperative not only to improve the understanding of the mechanisms behind these incurable disorders, but also to accelerate drug discovery [32].

Currently, there is a heavy reliance on animal models to study in vivo cell behaviour. They are regarded as the gold standard, since they closely mimic the complexity of the BBB microenvironment and the individual diversity found in humans [34,35]. Nevertheless, it is necessary to consider the occurrence of species-specific biological differences, and thus, ultimately, their inadequate reproduction of human pathophysiology [32]. Given the existence of immunological, genetic and cellular variances between humans and animals, no in vivo model is capable of faithfully recapitulate all aspects of human brain disease [36,37]. This is an important disadvantage, since it becomes problematic to translate the results obtained using animals to the human context. Indeed, more than 80% of compounds that were successfully tested in animal models failed in clinical trials [37]. Poor experimental methodology and species-to-species variability in protein expression profiles may also contribute to explain this issue [35]. Furthermore, it is believed that most NDs are polygenetic, comprising multiple, and frequently unrecognized, genetic alterations, which makes the generation of transgenic animals very challenging [38]. Other critical drawbacks of using animal models include ethical concerns, elevated costs and prolonged, low-throughput, labour-intensive processing [39]. The global implementation of the 3Rs principles (reduction, refinement and replacement) also underlines the requirement to abandon animal testing as much as possible in the future, rerouting even more the investigation of NDs pathology to in vitro models [32].

### 3.2. In Vitro Models

**Figure 2:**
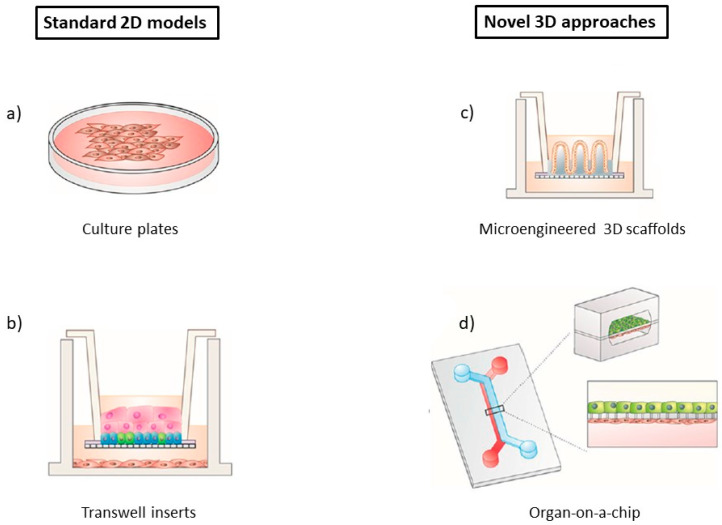
Schematic representation of different 2D and 3D in vitro models: (**a**) culture plates, (**b**) Transwell inserts, (**c**) microengineered scaffolds, (**d**) organ-on-a-chip. Reprinted and adapted with permission from Torras et al. [44].

To circumvent the issues regarding the use of animals, scientists commonly resort to two-dimensional (2D) and three-dimensional (3D) in vitro models as an alternative (Figure 2) [34]. Cell culture technology is fairly straightforward and can be performed in carefully monitored conditions, thus making it relatively robust and with good reproducibility [34,37]. Additionally, it is generally cheaper to acquire and maintain when compared to animal models [34]. There are different models available, namely, 2D or 3D static cell cultures, and 2D or 3D perfusion microfluidic systems [40].

Starting with 2D static cell cultures, they are a staple for many applications like toxicity or metabolism analysis [38,41]. These systems are well known, with extensive literature allowing to critically analyse the data obtained, and understand the cell behaviour in many conditions [40]. In the most basic 2D models, cells are attached to a medium-containing flat surface such as flasks, dishes or well plates, where they are able to adhere and spread [32,42]. To enable BBB drug transportation studies, the Transwell^®^ setup was created, with one or more types of BBB cells being cultured on semi-permeable microporous inserts [35,37]. Transwell^®^ systems are widely utilized, since they are user friendly and cost-effective, increasing test speed and cutting down reagent consumption [37]. Cells of human origin can be used, therefore sidestepping problems with translation of results to the clinic.

However, even when co-culture is done, 2D cell cultures have significant shortcomings. The production yield is low, and cell growth occurs unnaturally in reaction to external factors [42]. More importantly, there is a failure to properly replicate and reproduce key aspects of the BBB microenvironment, including cell–cell and cell–ECM interactions that occur within the NVU [34,41]. In addition, they are restricted in supplying suitable electrical, mechanical and chemical cues, which can lead to subtle differences in barrier permeability and cell morphology when compared to in vivo models [41]. Even though the realism of 2D cultures can be somewhat improved by incorporating more cell types or by using human cells, robustness is sacrificed, and thus, these models are not appropriate when the intention is to model higher order features and trajectories of the CNS [38].

3D cell systems have garnered attention for decades, being introduced as an attempt to better represent the intricate human physiology and relevant conditions, therefore allowing to improve the simulation of the in vivo setting of cells inside organs and tissues [32,41]. Traditional 3D cultures are usually achieved by culturing cells on biocompatible extracellular scaffolds (such as hydrogels), either in well plates or on Transwell^®^ membranes [32,34]. By using hydrogels that incorporate natural molecules of the ECM (e.g., collagen or fibrin) or synthetic polymers (e.g., polylactic acid (PLA) or polyglycolic acid (PGA)), cells are induced to polarize and interact with neighbouring cells, randomly interspersing in the ECM or self-assembling into physiological-like microstructures (organoids) [32,34,43]. Furthermore, culture systems can also self-organize in 3D aggregates in a scaffold-free manner, without the need of a supportive matrix, giving rise to spheroids [44].

Nonetheless, 3D cell cultures also have their own drawbacks. Firstly, some of these models are not high-throughput or cost-effective, and often, there are problems regarding the existing protocols not being reproducible [32]. The macroscale of 3D leads to challenges in the transportation of nutrients and oxygen, as well as in cell viability, analysis, sampling and imaging, as its complexity increases [41]. Although 3D in vitro cultures are better at emulating certain aspects of the spatial complexity of the CNS and of the BBB microenvironment (including molecular and signalling pathways), many systems still lack the level of multiscale architecture and tissue-tissue interfaces that influence the function and development of organs in both health and disease, including the brain: for instance, just like it happens with 2D models, cells in traditional static 3D models are not exposed to dynamic stresses like fluid flow from pressure gradients, or tension and compression. Moreover, they also fail to take into account biochemical effects that are specific to each cell type within a co-culture, when trying to reconstitute the NVU [10,32].

Recently, progresses in microfabrication techniques have allowed to the integration of cell/tissue engineering on microfluidic platforms, leading to the development of a new, revolutionary type of model: organ-on-a-chip. Organs-on-a-chip combine the advantages of in vivo and traditional in vitro models, while potentially overcoming most of their limitations [35,41]. In this sense, in the next sections we will discuss the value and the state-of-art of this highly promising approach to mimic BBB/brain physiology and function, as well as for studying NDs pathology.

## 4. Microfluidic BBB In Vitro Models (BBB/Brain-on-a-Chip)

The rise in microscale technologies has opened avenues for the development of more complex and advanced CNS biological models that are unachievable with conventional cell culture systems [39]. Organs-on-a-chip are microfluidic devices for culturing cells in continuously perfused, micrometre-sized chambers, engineered in such a way to enable them to closely mimic and better replicate the dynamic conditions, and the in vivo microstructure/environment of a tissue or organ (in this case, of the human BBB or brain) [10,35,45]. The goal is not to build a whole living organ but rather to synthesize minimal functional units that recapitulate tissue- and organ-level functions, thus allowing the development of a BBB model on a microdevice in order to study physiological and pathological phenomena [10,32,45]. Early research focused on simpler systems, consisting of a single microfluidic chamber containing one kind of cultured cell [46]. As knowledge advanced, more complex designs were created, generally entailing multiple microchambers connected with microchannels or microgroove arrays, and cultured with distinctive cell types to reproduce interfaces among different tissues (e.g., the BBB) [10,46].

Microfluidics-based models have the ability to regulate primary factors like cell patterning, cell–cell and cell–ECM interactions or transcellular oxygen, molecular and chemical gradients, as well as to provide physiologically relevant levels of fluid flow shear stress and cyclical mechanical forces analogous to those seen in blood vessels (i.e., perfusion culture) [10,41,46]. Accurate control of fluid flow is very useful, since it improves the function, differentiation and long-term survival of many cell types [10]. Such interactions and clear interplay between microenvironmental elements and culture parameters are not achievable with traditional cell systems [47]. Moreover, microfluidic approaches offer a plethora of other advantages that make them a unique platform to replace or at least complement other models for high-throughput drug screening and disease modelling, such as:

(1) Being highly customizable and having better experimental flexibility and control (such as in-chip design or in vitro biochemical and mechanical microenvironment conditions), thus giving reply to specific requirements of each experiment and cellular system [32,39,47].
(2) Significant reduction in chemical cost and consumption, since far less minute amounts of cells, culture medium and other reagents are needed [41,47].
(3) Continuous supply of nutrients and waste removal [40].
(4) Schedule flexibility [40].
(5) Reduced risk of contamination [47].
(6) Possibility of multifunctional integration of analytical biosensors and other electronic apparatus (e.g., microscopy devices, microelectrode arrays (MEAs), etc.) for real-time/on-chip or downstream monitorization of cellular behaviour, detection of physiological parameters (e.g., biomarkers or chemokines levels) and in situ analysis of external stimuli, therefore decreasing analysis time [32,40,46,47].
(7) High automation capability [47].
(8) Possibility of single-cell handling, compartmentalized culture, co-culture and long-term culture [39,47].
(9) Possibility to test in the same controlled environment both healthy and disease tissues [35].

The real value of microfluidics is their ability to independently vary many of the previously stated control parameters, while carrying out real-time, high-resolution imaging of the events at a molecular scale in the tissue or organ studied [10]. It is not surprising that the implementation of microplatforms has gained traction, being progressively employed in BBB studies, since they can closely simulate the in vivo microenvironment of the CNS, while using different types of cells (including neurons, glial cells or even brain tissues) [39]. Microfluidic systems replicate organ-level functions, being therefore better suited when compared with conventional in vitro models for higher level studying of biological interactions and response to stimuli within the BBB, cellular activities/mechanisms that play a key role in NDs pathology or even evaluation of CNS drugs [35,37,40].

Different manufacturing processes are available to control the surface topology, and construct organs-on-a-chip: photolithography, soft lithography, embossing, moulding, etching, 3D printing, laser ablation and so forth [38,39,47,48]. Appropriate selection of chip material is pivotal, since it can not only improve sensitivity, accuracy and stability, but also reduce experimental costs [37]. The most prevalently used material is polydimethylsiloxane (PDMS). PDMS is a biocompatible polymeric compound, which is reasonably inexpensive and can be applied in such a way to meet specific experimental requirements. It is robust and non-toxic, gas, vapour and water-permeable, and optically transparent, all important and convenient features for cell culturing in microfluidic chips. Furthermore, the fabrication and set-up of PDMS-based systems is relatively straightforward, with mass production of chips from one mould being easily attainable, which is especially advantageous in academia/university settings. However, PDMS devices have some limitations for BBB studies, such as incompatibility with most organic solvents or poor affinity for adhesion of living cells, given their hydrophobic nature. Moreover, their softness and elasticity make them less than ideal for industrial scale production. To overcome these constraints, thermoplastics (e.g., poly(methyl methacrylate), polystyrene, cyclic olefin copolymer) can be employed instead [37,48].

As indicated earlier, microfluidic systems can assume a 2D or 3D configuration [40]. Notwithstanding the design or configuration, when implementing a new microfluidic model it is always essential to evaluate that model’s performance [37]. This typically involves assessing barrier integrity and tightness by measuring transepithelial electric resistance (TEER) values, testing for the presence of specific BBB markers of TJs (e.g., zona occludens-1, claudin-5, P-gp) and observing the permeability of model compounds (e.g., fluorescein isothiocyanate (FITC) labelled dextrans or D-mannitol) [37].

Still, hurdles arise when trying to establish a culture on microfluidic devices [49]. Each key component of a BBB- or brain-on-a-chip (manufacturing method, chip material or design, culture biomaterials, cues and biosensors) can present specific technical challenges [48]. Manufacturing of these devices requires specialized micro-engineering skills [10]. The diversity of device designs, the lack of standardized culture protocols and of quantification of BBB parameters, the variety of cell lines, as well as the cell-specific responses/behaviour to microfluidic culture, make generalizations and comparison between platforms difficult [35,49]. Indeed, different cell types respond contrastingly when moved from macroscopic to microfluidic culture [49]. Furthermore, operational control is complex [49]: to achieve a consistent and robust cell seeding in the microchambers is tricky, as well as to precisely control the cell–cell and cell–ECM interactions necessary to generate exact tissue structure-function relationships [10]. The use of thin ECM coating or simplified ECM gels can lead to contraction or degradation of the matrix over time, which is obviously problematic for long-term cell survival, despite continuous perfusion. Occurrence of bubbles within the microchannels is another issue. Their complete removal can be difficult, potentially leading to cell damage [10]. Finally, satisfactory intra- and inter-batch reproducibility of the same organ-on-a-chip still needs to be achieved [48].

### 4.1. 2D Microfluidic Systems

Recently, Jeong et al. [50] devised a PDMS bilayer chip design wherein neural endothelial cells and astrocytes were co-cultured in a polycarbonate membrane. With this configuration, the endothelial cells and astrocytes are physically separated and grow in two separate microenvironments, being allowed however to establish localized interactions through the pores of the membrane, which is important for the development of a realistic brain-capillary interface. The cross-section of the 4 microchannel rows and columns that comprise the luminal and abluminal PDMS layers (intending to represent the blood and brain side, respectively), resulted in an array of 16 semi-independent BBB testing areas on a single chip. Each of the 16 BBB testing areas was fitted with integrated electrical sensors to analyse non-invasively and continuously the TEER values. Barrier function was additionally confirmed using two other methods, namely, immunofluorescent staining and molecular permeability assays. Immunostaining revealed that there was a time-dependent formation of TJs, since the expression of ZO-1 (a TJ protein) increased accordingly with the division of endothelial cells. Moreover, the authors demonstrated that the ZO-1 expression level is also affected by ECM composition and sheer stress. Regarding ECM selection, coating the membrane with Matrigel^®^ generated higher TEER values over time (615 ± 122 Ω at day 0 to 3368 ± 441 Ω at day 4) when compared to fibronectin coating, which correlates to the time-dependent increases in ZO-1 expression, and justifies the use of this material in subsequent experiments. Higher levels of sheer stress led to increased number of TJs, but when too high (30 dyn/cm^2^), ZO-1 expression actually decreased. Therefore, an optimized level of 20 dyn/cm^2^ was used, falling under the range of reported in vivo levels (5–25 dyn/cm^2^). Dextran permeability assays showed co-culture conditions have a permeability coefficient almost 2.5-fold lower compared to a monoculture of endothelial cells only, reinforcing the notion that the interactions between endothelial cells and astrocytes are essential for the formation of a tighter BBB barrier. Furthermore, the triple co-culture chip provided preliminary evidence of a closer, more faithful drug testing capability to the one that happens in in vivo BBB, since even after treatment with histamine (a known TJs disruptor), almost no change in the TEER values was detected, whereas for the monoculture BBB chip, there was a drop in the TEER values, which indicates an increase in permeability [50].

Griep et al. [51] further demonstrated that fluidic forces affect the integrity of the BBB. They showed that applying a physiologically relevant shear stress (5.8 dyn/cm^2^) on a small BBB-on-a-chip, composed of a monolayer of a human cerebral microvascular endothelial cell line (hCMEC/D3) cultured in a membrane coated with collagen I and two layered PDMS chamber, results in an increased expression level of TJ proteins, improving endothelial cell function and inducing a significantly higher barrier tightness. This resulted in a 3-fold increase of the average TEER value from 37 Ω·cm^2^ (static conditions) up to 120 Ω·cm^2^, measured with integrated platinum electrodes. Moreover, barrier function is also biochemically modulated, since stimulation with inflammatory protein tumour necrosis factor α (TNF-α) negatively impacted the TEER, leading to a 10-fold decrease, down to 12 Ω.cm^2^. The use of a single device to compare both static and dynamic TEER values is advantageous, given that it avoids possible experimental variability [51].

**Figure 3:**
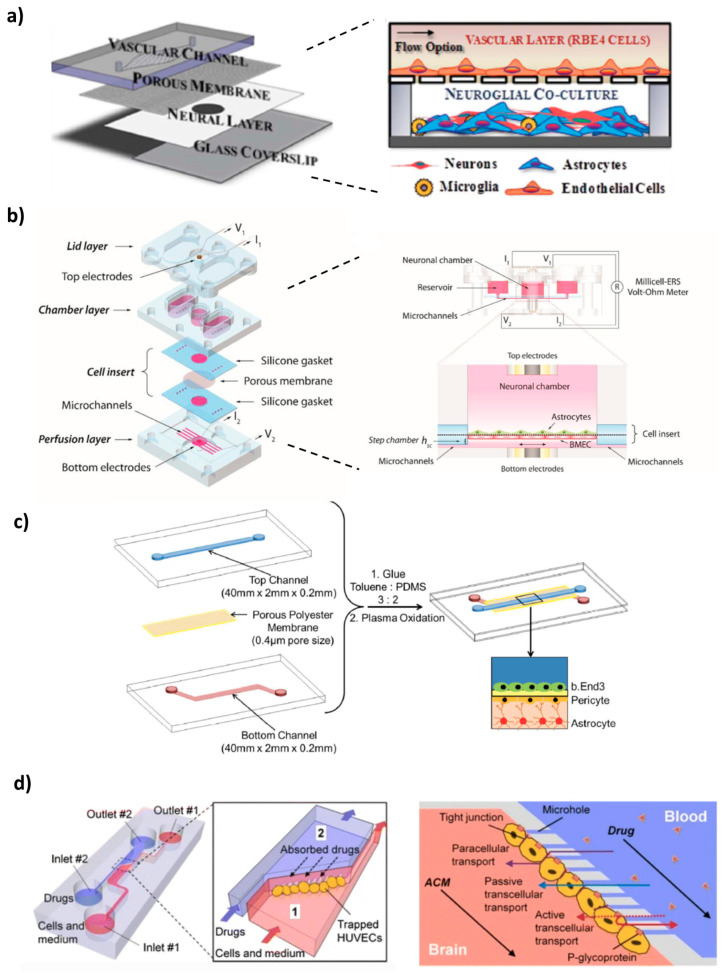
Examples of 2D BBB microfluidic models: (**a**) modular polydimethylsiloxane (PDMS) device to recreate the NVU by co-culture of neuroglial and endothelial cells, (**b**) pumpless microdevice that provides in vivo-like BBB properties for drug permeability screening, (**c**) multi-layered system composed of a triple culture of co-immortalized mouse brain endothelial cells (bEnd.3), mouse astrocytes (C8D1A) and pericytes, (**d**) microfluidic device that mimics the cerebral vasculature and is a reliable permeability assay system. Reprinted and adapted with permission from: (**a**) Achyuta et al. [52], (**b**) Wang et al. [53], (**c**) Wang et al. [55]. Copyright (2016) American Chemical Society, (**d**) Yeon et al. [56].

Achyuta et al. [52] made a modular device to recreate in vitro the NVU (Figure 3a). The device consisted of two PDMS parts—a vascular conduit overlaid on top of a neural chamber—, separated by a polycarbonate membrane. The vascular chamber was coated with bovine plasma fibronectin, and rat brain endothelial cells (RBE4 cell line) were cultured in this channel for 2 days under static conditions. In its turn, the neural part was coated with poly-D-lysine and cultured with E-18 rat cortical cells for 10 days, which differentiated into a mixture of astrocytes (95%), neurons (4%) and microglia (1%), in the presence of serum and basic fibroblast growth factor (b-FGF). Since brain endothelial and neuron–glial cells have different maturation periods, they were cultured separately, and afterwards, both chambers were assembled, in order to allow communication via the membrane. Functional assays were conducted. Both E-18 and RBE4 cells showed good viability (>90%) upon live/dead staining. Immunofluorescence revealed the presence of different markers such as von Willebrand factor (vWF), glial fibrillary acid protein (GFAP), microtubule-associated protein 2 (MAP-2) or OX-2, indicating proper neural and vascular cell differentiation. Furthermore, western blot showed the presence of TJ protein ZO-1. The integrity and tightness of the barrier was assessed with Alexafluor^TM^-conjugated dextran leakage, perfused during 1h through the vascular chamber at the rate of 1 mL/h. The models containing a RBE4 cell layer presented significantly decreased leakage into the neural reservoir compared to devices without cells. Conversely, when the vascular layer was exposed to TNF-α for 6h, more dye was leaked, indicating a pro-inflammatory reaction that disrupted barrier function and triggered BBB hyperpermeability. Moreover, the circulation of the inflammatory stimulus, triggered the activation of 75% of astrocytes and microglia in the neural chamber, mimicking neuroinflammation. Several caveats of the current model were pointed out by the authors, including the use of embryonic stem cells that do not reproduce all the features of mature in vivo NVU, the absence of pericytes and shear stress, or the rodent-provenience of the cells, which might make direct correlation with data from human clinical trials impossible. However, if needed, perfusion could be applied via the vascular channel, in order to mimic in vivo settings. In addition, control over each module also allows the user to easily manipulate cellular seeding and cellular:neuroglial ratios, to provide other physiological cues that impact the NVU and to observe sub-cellular features through high-content analysis. The system could be an opportunity to deliver nutrients, drugs, cells or nanomaterials, and evaluate their impact on the NVU [52].

In a 2017 study, Wang et al. [53] developed a 2D pumpless BBB microfluidic model (Figure 3b). BBB constructs were prepared by co-culturing, up to 10 days, brain microvascular endothelial cells (BMECs) (derived from human-induced pluripotent stem cells (iPSC)) with rat primary astrocytes, on two sides of a collagen IV and fibronectin-coated porous insert. The final assembled device was achieved by accommodating the insert between a bottom layer with microchannels (perfusion layer) and a middle layer containing a central neuronal chamber and reservoirs filled with culture medium. Recirculation was assured without the need of using tubing and pumps, by means of a rocking platform that changes the tilting direction. In order to properly mimic and match tissue volume and blood residence time of a human adult brain, the neuronal chamber and perfusion flow rate were scaled down proportionally (residence-time based design). Furthermore, to minimize oscillatory fluidic shear stress on the cell surface, a “step chamber” was introduced, increasing the distance between the cell plane and the perfusion layer. This modification enabled the BMECs to survive and maintain their unique BBB phenotype, while still providing physiologically relevant flow rates [53]. A TEER measuring probe with costum Ag/AgCl electrodes was integrated into the microfluidic device, facilitating the monitoring of the values and the optimization of culture conditions. This was the first microfluidic model to achieve sustained high TEER values (above 2000 Ω·cm^2^ for up to 10 days) that fall within the range of those recorded in vivo (1500–8000 Ω·cm^2^), being also the highest reported for any BBB-on-a-chip system (peak TEER above 4000 Ω·cm^2^). Likewise, evaluation of permeability coefficients for several large tracer molecules (4, 20 and 70 kDa FITC-dextran) and small drugs (caffeine, cimetidine and doxorubicin), strongly correlated with in vivo BBB transport data. Doxorubicin disrupted the BBB integrity after 24h treatment. The authors denote the convenience and simplicity of their setup, also indicating that since it closely mimicked BBB barrier functions, the potential and suitability of this approach for brain drug screening studies was validated. A possible improvement would be to incorporate astrocytes of human origin instead of rat, in order to create a fully human BBB model [53].

Walter et al. [54] recreated the BBB on a biochip that consisted of a polyethylene terephthalate (PET) membrane, situated between two PDMS channels fixated with a silicone sealant. This core was placed between glass slides with gold electrodes. Moreover, attached to the top glass slide, there were two PDMS blocks with reservoirs holding culture medium. The upper PDMS channel was defined as the “blood side” and coated with collagen I, while the lower channel, representing the brain, was coated with collagen IV. The presence of collagen, a natural element of the ECM, facilitates the seeding of cells. This versatile microdevice enabled the formation of two different BBB models: hCMEC/D3 human brain endothelial cell line and triple co-culture of primary rat brain endothelial cells with primary astrocytes and brain pericytes. The endothelial cells were cultured on the top side of the PET membrane in the top channel, while pericytes and astrocytes were cultured on the bottom channel. For 3 days, the cells were grown under static conditions, after which a peristaltic pump provided dynamic culture conditions at low shear stress. Characterization of cell culture was done by assessing its morphology, TEER values and apparent permeability. As expected, the results demonstrated that the primary rat triple co-culture was more efficient in the induction of barrier properties: it exhibited higher TEER values (114 ± 37.5 Ω·cm^2^ for both static and dynamic culture conditions vs. 19 ± 2.8 Ω·cm^2^ and 28.5 ± 7.2 Ω·cm^2^ for the hCMEC/D3 under static and dynamic conditions, respectively) and lower tracer permeability coefficients, indicating a tight barrier. Furthermore, immunostaining and confocal microscopy confirmed a stronger expression of both β-catenin (adherens junction protein) and ZO-1 (TJs protein) for those cells [54].

Likewise, a multi-layered BBB microfluidic model, composed of a triple culture of co-immortalized mouse brain endothelial cells (bEnd.3), mouse astrocytes (C8D1A) and pericytes, seeded on a 0.4 µm micropore membrane fabricated using soft litography, was developed (Figure 3c) [55]. The membrane was integrated between two partially overlapping PDMS microchannels, embedded with Ag/AgCl electrodes. The top channel was connected to a pump that provided the culture medium, exposing the endothelial cells to a fluidic shear stress of 1.6 dyn/cm^2^. A live/dead cytotoxicity assay proved a high viability of all cells up to 21 days, which was further confirmed by fluorescent images of their morphology. Interestingly, it was shown that cell alignment is influenced by the microchannel’s size and shape, since the spindle of the cells gradually decreased along the longitudinal axis of the channel as a function of days in culture. Compared to a single bEnd.3 monolayer and a double endothelial cells-pericytes co-culture, the triple model had enhanced functional expression and activity of P-gp, given that the basolateral-to-apical permeability and the efflux ratio of dexamethasone (a substrate for P-gp) were superior, gradually increasing with the increase in culture time, thus underlying the functionality of the efflux pump in the endothelial cells. Furthermore, it showed higher TEER values. It is known that soluble factors such as TGFβ, bFGF or GDNF, secreted by astrocytes and pericytes, can have a positive effect in reinforcing the integrity and barrier properties of the BBB model. The authors demonstrated that increasing the height of the lower channel from 200 µm to 600 µm and 1000 µm, translated in a progressive decrease of the TEER values. As channel height increases, so does the medium volume, resulting in a dilution of those factors and, therefore, in a reduced restrictiveness of the formed barrier. Permeability screening assays with [14C]-urea and [14C]-mannitol showed that both the bi- and triple co-cultures have size selectivity, being able to discriminate between the two different markers. Moreover, permeability of [14C]-mannitol after 18 days in the triple culture was similar to its reported permeability across the BBB in vivo [55].

Yeon et al. [56] designed a BBB microfluidic model comprised of a PDMS chip with two channels, connected by microholes (Figure 3d). Pressure gradients generated by applying different flow rates in the microchannels, led to hydrodynamic entrapment of human umbilical cord endothelial cells (HUVECs) in the microholes. Within 2h of incubation with astrocyte-conditioned medium (ACM), a barrier was formed, and the permeability of various FICT-tracer dextrans (4, 40, 70 kDa) and drugs (antipyrine, carbamazepine, atenolol, verapamil and propranolol) through the HUVEC layer was evaluated by fluorescence microscopy and high-performance liquid chromatography (HPLC), respectively. Compared with the untreated control, supplementation with ACM significantly decreased the permeability of the dextran dyes, as well as of carbamazepine, verapamil, antipyrine and atenolol, which is in good agreement with the enhanced expression of ZO-1 TJ protein detected by immunofluorescent staining. In the case of propranolol, the difference in permeability was negligible. Moreover, these values highly correlate with the results measured in conventional Transwell^®^ systems, as well as with in vivo permeability data, validating the reliability of this model to predict the penetrability of new CNS-targeted molecules. The authors also confirmed that exposure to hydrogen peroxide, an inductor of ROS formation, contributed to ZO-1 redistribution to the cytosol, therefore increasing BBB permeability. Two of the main concerns of this device are that it lacks cell–cell contact, a fundamental characteristic of the BBB in vivo, and it does not reproduce the in vivo dimensions of microvasculature [56].

As it is perceptible from the studies described so far in this section, one of the many applications of microfluidic platforms is the assessment of the permeability of different compounds, facilitating the early screening of brain-targeted drug candidates [7,37]. This is not only applicable to conventional dosage forms, but also to novel biopharmaceuticals and nanomedicines [7]. In regard to this last category, Papademetriou et al. [57] carried on a study wherein the objective was to evaluate the impact of static and flow conditions on the BBB binding and internalization of angiopep-2 coupled liposomes. The microfluidic device consisted of two S-shaped PDMS microchannels, with the region of overlap being separated by a polycarbonate membrane treated with a mixture of fibronectin and collagen IV, wherein mouse brain endothelial cells (b.End3) were cultured in the upper part over 3–6 days prior to experiments. The liposomes (mixture of 1,2-dipalmitoyl-sn-glycero-3-phosphocholine (DPPC), 1,2-distearoyl-sn-glycero-3-phosphoethanolamine-N [methoxy(polyethylene glycol)-2000 (DSPE-PEG2k) and DSPE-PEG maleimide, MW 3400 (DSPE-PEG-MAL3.4k)) were prepared by lipid film hydration method [58], followed by angiopep-2 conjugation. Angiopep-2 (ang-2) is a peptide of the LPR1 transferrin receptor, facilitating brain transport and penetration. The formation of a functional barrier was confirmed by dextran permeability size selectivity assays, as well as the measured TEER value (172 Ω·cm^2^), which is far superior to baseline values already reported for BBB microfluidic models using bEnd.3 cells (30–50 Ω·cm^2^). Results from fluorescence microscopy and spectroscopy allowed to withdraw two main conclusions: firstly, that the process of functionalization resulted in significant binding to bEnd.3 cells compared to non-functionalized liposomes, and secondly, the ang-2 nanocarriers were less internalized by the endothelial cells via receptor-mediated transcytosis under high shear stress (6 dyn/cm^2^) relative to low flow shear stress (1 dyn/cm^2^) or in static conditions. This suggests luminal fluid flow impacts the binding levels, and that at higher flow shear stress, detachment forces resulting from the flow are enough to overcome the avidity of ang-2 liposomes, uncoupling them from the endothelial cells. The claudin-5 perinuclear expression hints that flow exposure might have partially disassembled TJs, which may have influenced the penetration of NPs through the model. Since physiological flow shear stress in brain capillaries ranges from 5–23 dyn/cm^2^, which is important to replicate, tuning the NPs characteristics (e.g., ang2 valency) might help to solve this problem by enabling binding in the presence of flow while maximizing the BBB penetration. As mentioned by the authors, one limitation of this study was the lack of co-culture with other cell types that form the NVU [57]. In addition, Park et al. [59] characterized the transcellular transport of angiopep-2 quantum dot NPs and monoclonal antibodies directed against the transferrin receptor. The results of this study suggest that hypoxia is needed to better recapitulate the native BBB in terms of transferrin receptor function, as well as of other BBB transporters, since when compared to normoxic conditions, hypoxic conditions resulted in enhanced differentiation and increased expression of those proteins [59].

In similar fashion, the effectiveness of a gH625 (a membranotropic peptide) to direct the transport of polystyrene NPs across a BBB layer under flow conditions that mimic circulation, was tested in a microfluidic device devised by Falanga et al. [60]. Its design was based on the thermoplastic polymer poly(methyl methacrylate) (PMMA), which has the advantage of being easily micromachinned by micromilling. Confocal microscopy and phase contrast demonstrated the formation of a confluent bEnd.3 layer on a porous membrane, at 7 days of culture. Crossing experiments proved that functionalization with gH625 peptide enhanced the adhesion of the NPs to the BBB layer, and at a working medium flow rate of 5 µL/min, increased 2-fold the transport of NPs (6.13% compared to 2.72% of non-functionalized blank NPs). Furthermore, TEER analysis confirmed the maintenance of barrier function before and after the passage of the nanocarriers, proving that the bEnd.3 layer is not disrupted by the NPs. In summary, the system allowed the reproducibility of experiments and the quantification of NPs transport across the BBB in vitro [60].

Prabhakarpandian et al. [61] introduced a synthetic microvasculature model of the BBB (SyM-BBB). The device consisted of a circular PDMS chip with microchannels, partitioned in a two-compartment chamber: an outer ring (blood compartment—apical side) and an inner ring (brain compartment—basolateral side), which were separated by an array of micropillars with 3 µm gaps between them. These gaps allowed communication between the chambers. The outer ring was coated with fibronectin, and RBE4 rat endothelial cells were cultured under fluidic shear conditions (0.1 μL/min) for 24-48h. ACM was added to the brain compartment, and its influence on barrier permeability, tight junction formation and P-gp expression/activity was studied. Compared to a conventional Transwell^®^ and a device without ACM, the ACM-perfused model improved the overall BBB function. It had decreased barrier permeability (reflected by lower FITC-dextran intensity levels in the basolateral chamber), promoted expression of TJ proteins (ZO-1 and claudin) and P-gp, and increased efflux pump activity (translated in a significantly higher efflux of Rhodamine 123, even in the presence of verapamil, an inhibitor of P-gp). One thing that differentiates this model from the other two-chamber assays that were described earlier is that it does not use a membrane or a filter, with the apical and basolateral compartments being side-by-side, which simplifies fabrication. Furthermore, its design allows the simultaneous, real-time monitoring of both compartments, provides realistic microvasculature environment and dimensions, delivers physiological shear stress/fluid flow and allows long-term cell culture [61].

### 4.2. 3D Microfluidic Systems

Marino et al. [62] resorted to a two-photon lithography technique (3D printing) to fabricate a 3D microtubular porous structure that replicates the microcapillaries of the NVU. The core benefit of this technique is that capillary diameter, pore size or pore density are easily fine-tuned. The structure consisted of 50 parallel microtubes with pores on the surface to allow transport toward the external environment, connected to an inlet and an outlet reservoir. The microtubes served as scaffolds for the co-culture of murine brain-derived endothelial cells (bEnd.3) and U87 glioblastoma line, allowing the cells to maintain their morphology and phenotype, since it mimics the basement membrane of the BBB. Culture medium was pumped at 50 µL/h, making the flow rate within each microtube of 1 µL/h, a value comparable to that of cerebral microcapillaries. The novelty of this work is that the platform was constructed according to the actual dimensions of human brain capillaries, leading to the first-time development of a real scale, biohybrid and biomimetic BBB model that showed an efficient maturation of TJs (high expression of ZO-1 by immunofluorescence staining), decreased dextran transcytosis and higher TEER value (75 ± 2 Ω cm^2^), with respect to the same microfluidic system without cells. Even after 5 days, TEER value remained stable (71 ± 10 Ω cm^2^), denoting preserved cell viability and functionality. The investigators pointed out that this model could potentially be used for high-throughput screening of drugs or nanomaterials for several brain pathologies [62].

Wevers et al. [63] established a perfused parallel BBB-on-a-chip model, comprised of a two- or three-lane microfluidic platform that harbours 96 or 40 chips, respectively, in a 384 well plate format (referred to as OrganoPlate). In each chip, a microvessel of brain endothelial cells (TY10 cell line) was formed and grown against an ECM gel composed of collagen-I. On the other side of the gel, astrocytes (hAst human cells) and pericytes (hBPCT human cells) were added, in order to complete the model. By placing the plate on a rocker and infusing it with medium culture on the microvessel channel, perfusion was generated, creating a bidirectional flow from inlet to outlet and vice-versa. Expression and interendothelial localization of key BBB TJs (claudin-5) and adherens junction (VE-cadherin, PECAM-1) proteins was detected by immunostaining, indicating barrier formation. Moreover, barrier integrity was assessed at days 5, 7 and 9 by a fluorescent permeability assay. The results showed that the model was able to severely restrict the passage of a 20 kDa FICT-dextran dye, which is revealing of a functional BBB. To study BBB diffusion by receptor-mediated transcytosis of a therapeutic antibody (antibody to human transferrin receptor (MEM-189)), the authors infused the microvessel channel with MEM-189 or a control antibody, quantifying afterwards its levels in the basal chamber. The penetration of MEM-189 was approximately 2-fold higher than the penetration of the control antibody (apparent permeability of 2.9 × 10^−5^ versus 1.6 × 10^−5^ cm/min, respectively), denoting that the binding of the therapeutic antibody to the transferrin receptor expressed in the endothelial cells facilitates the transportation across the BBB. All in all, the Organoplate has several advantages such as minimal absorption, low-level passive permeability and ease of sampling, that make it a valuable and suitable model for high-throughput drug screening (including of large molecules like antibodies) under physiological conditions without the need of artificial membranes [63].

**Figure 4:**
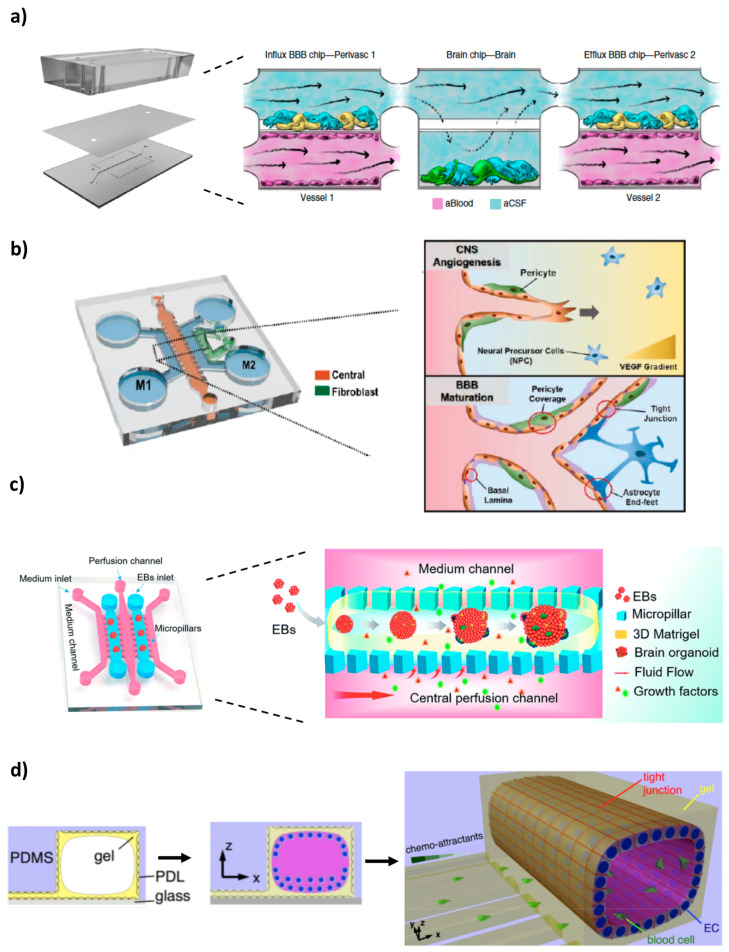
Examples of 3D BBB microfluidic models: (**a**) three interconnected systems—one brain chip between two BBB chips—to obtain insights into previously unknown metabolic interactions between the different cells of the NVU, (**b**) 3D brain angiogenesis model for reconstitution of BBB efflux transporter system (P-gp) under perfusion conditions, (**c**) brain organoid-on-a-chip that recapitulates the early human brain development, (**d**) microfluidic platform for the study of neurovascular pathology. Reprinted and adapted with permission from: (**a**) Maoz et al. [64], (**b**) Lee et al. [75], (**c**) Wang et al. [76]. Published by The Royal Society of Chemistry, (**d**) Cho et al. [78].

Maoz and colleagues [64] recently constructed an innovative and sophisticated platform of three interconnected microfluidic systems (Figure 4a). The modular structure comprised a brain chip put between two BBB chips, thus recapitulating at the same time the brain parenchyma and the influx/efflux across the BBB. The brain chip contained human neural stem cells (~60% glial cells and 40% of dopaminergic, glutamatergic, serotonergic and GABAergic neurons) and astrocytes cultured on the laminin and poly-l-lysine-coated surface of the lower compartment, whereas the BBB chips entailed a monolayer of primary human brain microvascular endothelial cells (hBMVECs) at the vascular (lower) chamber and astrocytes and pericytes at the perivascular (upper) chamber, to mimic the external wall of a brain microvessel. To achieve successive influx and efflux from one chip to the other, and to allow diffusion-mediated molecular transport to dominate (as what happens in vivo in the brain), the shear stress was adjusted to be close to 0 within the brain compartment. The results of proteomic analysis demonstrated that fluidic coupling modulates the phenotype of the cultured cells via paracrine signalling, with more effective expression of some proteins (e.g., metabolism-associated proteins) in the coupled chips compared to uncoupled cultures. Furthermore, the linked system allowed to compare the individual roles of the different endothelial, neuronal and perivascular cell populations in the NVU, obtaining insights into previously unknown metabolic interactions between them that are significant for the maintenance of brain function. Of relevance, these metabolic interactions led to an enhancement of the synthesis and secretion of neurotransmitters gamma aminobutyric acid (GABA) and glutamate, suggesting that brain vasculature may have a role in underlying mechanisms of NDs. To investigate the drug screening capabilities of the model, the neuroactive drug methamphetamine was introduced in the BBB influx vascular channel. Methamphetamine produced a reversible disruption of the barrier, since removing the compound from the system stopped its effects that were leading to BBB breakdown, like reduced expression of cell–cell junction proteins (e.g., VE-cadherin). This microfluidic model can, therefore, be used for diverse applications, such as improving the basic knowledge on brain pharmacokinetics, facilitating the evaluation of penetrance, efficacy and toxicity of CNS-targeted drugs, or assessing BBB function and mechanisms in both normal and diseased states [64].

Bang et al. [65] established a 3D BBB microfluidic model, based on a vasculogenesis-like process. The device consisted of a PDMS slab containing one middle chamber and two channels side by side (vascular and neural channel). A vascular network self-assembled in the middle channel on a fibrin hydrogel over a 3-day period, by co-culturing human umbilical vein endothelial cells (HUVEC) and primary human lung fibroblasts, while in the adjacent micropost traps, astrocytes and rat cortical neurons were grown in direct contact with the capillaries, in order to complete the BBB model. The two channels allowed to independently supply different types of media for the neural and endothelial cells (neurobasal medium and endothelial growth medium, respectively), delivering the best combination of medium. Thus, an optimized co-culture environment not only increased cell viability, but also led to further inducement of relevant BBB characteristics, including greater expression of TJs protein (e.g., ZO-1), presence of synaptic structural features and a higher degree of neurovascular interfacing (establishment of more direct connections between astrocytic end-feet and endothelial cells). Correspondingly, this model had low permeability coefficients for 20 and 70 kDa dextran, comparable to reported in vivo BBB values [65]. While the developed platform is great for improving tissue-level function, presenting substantial potential to majorly accelerate brain disease neuropharmacological research, it cannot be considered a complete NVU model, since more cells would need to be included [65].

Partyka et al. [66] investigated in their 3D model the effects of mechanical stimuli exerted by blood flow on both BBB permeability and waste transport. The device consisted of two parallel microchannels with inlets and outlets, and a hydrogel at the centre of the microchannels. The hydrogel, composed of hyaluronan, collagen type I and Matrigel, supported the co-culture of human cerebral microvascular endothelial cells (hCMEC/D3) with astrocytes, for 3-4 days. As shown in previous studies [51,67], the findings suggest that both shear stress and cyclic strain increase TJs formation and decrease transendothelial permeability, significantly improving the overall BBB integrity. Accordingly, compared to static controls, vessels exposed to fluid flow, with or without stretch, had enhanced TEER values and lower permeability coefficients for dextran 4 kDa, thus confirming that these factors indeed contribute to the maintenance of proper BBB function. It was also demonstrated that vessel wall pulsation provides a convective force that facilitates retrograde transport along the basement membrane of the cerebral microvasculature, including of metabolic waste products. The authors concluded their study by postulating that attenuated pulsatile waste transport as a consequence from stiffening of the vessel walls, can possibly contribute for the pathogenesis of brain diseases [66].

Adriani and collaborators [68] developed a 3D hydrogel-based NVU system. The PDMS device was composed of four distinct parallel channels, where the first provided neural cell culture medium, the two central channels were used to co-culture astrocyte and neuron rat cells embedded in adjacent collagen I matrices, and the remaining fourth hosted cerebral endothelial cells (either HUVEC or hCMEC/D3) to mimic the blood vessel wall and provided endothelial cell culture medium. Immunochemistry assays revealed that all three cell types were capable of growing, displaying type-specific markers (e.g., presence of doublecortin (DCX) for neurons, GFAP for astrocytes and VE-cadherin or ZO-1 for the endothelial cells) and morphological characteristics. Permeability assays for dextran 10 and 70 kDa were done in order to assess barrier functionality. The results showed that co-culture with astrocytes enhanced BBB integrity, and that while both HUVEC and hCMEC/D3 monolayers demonstrated size-selective penetrability, the hCMEC/D3 line had significantly higher barrier integrity compared to HUVEC. Non-inclusion of a porous membrane within the system allowed a closer association between astrocytes end-feet and the endothelial cells, leading to a low permeability. The presence of astrocytes was also important for neuron growth, as demonstrated by the increased number of neurite segments in co-culture conditions, compared to neurons in monoculture. To best demonstrate the model’s BBB restrictiveness and its application for compound testing, the authors conducted a test in which glutamate, a neurotransmitter, was injected into the endothelial cell channel and its ability to trigger neuronal activity was assessed by calcium imaging and c-Fos expression. In contrast to a microfluidic system without endothelial barrier, the developed model had a significant decreased calcium concentration in the neurons and a C-Fos immunopositive staining, implying the presence of an intact barrier that was able to restrict the passage of the test compound and thus, consequently, restrict neuron activation. It is suggested that this platform will be useful for neurovascular studies, such as assessing drug effects on neural function [68].

Campisi et al. [69] co-cultured human induced pluripotent stem cell-derived endothelial cells (hiPSC-ECs) with human primary astrocytes and pericytes in a microfluidic platform. The mixture of cells was injected with a fibrin gel to the central microchannel of a PDMS device. The hiPSC-ECs underwent self-assembly in the 3D matrix into a microvascular network comprised of small perfusable lumens, with pericytes and astrocytes surrounding and adhering to them, thus emulating the in vivo BBB neurovascular organization. Immunochemistry and real time RT-PCR assays, performed after 7 days, validated the formation of a functional barrier, with gene expression of several BBB membrane transporters (e.g., P-gp, MRP1 or GLUT-1), TJs proteins (ZO-1, occluding and claudin-5) and ECM proteins (laminin and collagen V) in the triple co-culture being significantly increased compared with an iPSC-ECs monoculture or an iPSC-ECs/astrocytes co-culture. This BBB model was robust and physiologically relevant, displaying low permeability and transport selectivity dependent upon molecular weight. Indeed, permeability values of 2.2 × 10^−7^ cm/s and 8.9 × 10^−8^ cm/s for 10 kDa and 40 kDa FTIC-dextran, respectively, were similar to the levels measured in rat brain, as well as previous in vitro BBB microfluidic systems that also employed iPSC-ECs in co-culture with astrocytes or neurons. Astrocytes and pericytes work together through paracrine signalling and juxtacrine interactions to facilitate and stabilize endothelial vasculature organization and improve BBB formation/integrity, therefore explaining the fact that the triple co-culture showed the best performance in terms of stability and permeability. Furthermore, it was observed that the seeding of additional iPSC-ECs on the side channels improved perfusability and decreased permeability, by means of filling eventual gaps, increasing the number of connections in the vascular network and improving the overall coverage on the boundaries of the gel region. The authors have stated that the developed model could be applied for a number of applications, such as the study of angiogenesis and NVU function, investigation of BBB transcytosis, or evaluation of biological events that occur in NDs and of metastatic cancer extravasation to the brain [69].

More groups have also come up with recent 3D BBB microfluidic models using human iPSC [70,71,72]. Jamieson et al. [70] co-cultured iPSC-derived BMECs and pericytes in a cylindrical channel surrounded by collagen I. They observed the formation of a confluent monolayer without discontinuities, direct BMECs-pericyte contact and abluminal localization of the pericytes. The permeability of Lucifer yellow for the co-culture was comparable to that of BMECs microvessels without pericytes [70]. This contrasts with other BBB studies, where pericyte co-culture improved the barrier restriction [69,73,74]. These discrepancies may be due to a series of factors, such as variability in the culture and assay conditions, or even iPSC-line background. Linville et al. [71] cultured iPSC-derived BMECs within collagen I-coated microchannels in a PDMS microfluidic chip, leading to the creation of a model that resembles human brain post-capillary venules in terms of their cylindrical geometry, cell–ECM interactions and shear flow. This 3D perfused microvessel model had similar restrictive permeability to post-capillary venules in rats. Moreover, in comparison with self-organizing BBB approaches employed to mimic vasculogenesis, this model has the advantage of achieving physiological barrier functionality after only two days of culture, without the hassle of astrocyte or pericyte co-culture [71]. Motallebnejad et al. [72] created a BBB-on-a-chip that supports flow and where a co-culture of hiPSC-derived BMECs with astrocytes embedded in a 3D hydrogel was achieved. Apical addition of TGF-β1 conducted to a reduction of TEER values and astrocyte activation, putting forth the ability of the system to be used as a BBB disruption model. Furthermore, since all the cultured cells derived from the same hiPSC line, it could also enable genetic and rare disease modelling [72].

To get further insights into the BBB system of efflux transporters, which is generally not the main focus of in vitro brain platforms, Lee et al. [75] established a CNS angiogenesis microfluidic model containing a co-culture of human HBMECs, astrocytes and pericytes in a fibrin/hyaluronic acid hydrogel, seeded in the central microchannel (Figure 4b). Fibroblasts were also added to a side channel, as a source of angiogenic factors. Confocal images showed that after 7 days, the microvasculature resulting from the triple culture was fully mature and perfused, presenting physiologically relevant BBB characteristics, including in vivo 3D-like morphological phenotypes, direct cellular interactions (astrocyte and pericyte covering the vessels), increased adherens/TJs expression and limited vessel dilation, related to the minimized vessel diameter of about 34.64 μm (the smallest engineered vessel diameter ever reported). Additionally, compared to a monoculture of ECs, the triple co-culture had lower permeability for both 10 and 70 kDa FITC-dextran, which further confirmed the integrity and barrier function of the vascular network. To demonstrate the functionality of efflux transporters, the authors conducted a calcein acetoxymethyl (calcein-AM) assay, again under mono- and triple-culture conditions. Calcein-AM is a compound that when inside the cells is rapidly hydrolysed to fluorescent calcein (leading, therefore, to an increase in fluorescence). The developed model had a significantly lower initial calcein intensity, indicating a higher expression of efflux transporters that pumped out more calcein. Furthermore, fluorescence intensity on the endothelium was tracked over a 10h period, after treatment with P-gp inhibitors (valspodar and elacridar). As expected, for both culture conditions, the remaining fluorescence intensity was higher, since less calcein was being effluxed. However, compared with monoculture conditions, the triple co-culture had a more prominent difference between treated and non-treated groups. Under inhibitor treatment, the effect of efflux transport was diminished, leading to a higher remaining calcein intensity than in non-treated groups (8.96- and 2.10-fold higher for valspodar and elacridar, respectively). This was the first in vitro BBB model that allowed reconstitution and regulation of the efflux transport system under 3D microvasculature condition, highlighting its potential for application in CNS drug penetration studies [75].

In an interesting study, Wang et al. [76] converged two approaches–organoids and microfluidic devices-to develop a 3D brain organoid-on-a-chip that recapitulates the early human brain development (Figure 4c). The culture of hiPSCs-derived embryoid bodies (EBs) in a Matrigel^®^ within the microdevice, under mechanic perfusion flow, led to the in-situ growth and maturation of self-organized organoids. The microfluidic organoids exhibited well-defined neural differentiation, regionalization and cortical organization. Moreover, compared to brain organoids under static culture conditions in a Petri dish, the on-chip organoids had superior expression of cortical layer markers (TBR1 and CTIP2) and increased cell viability (<10% apoptosis vs. 40% apoptosis), indicating the importance of perfusion flow and 3D ECM in enhancing brain organogenesis and creating a biomimetic microenvironment that supports prolonged culture. The main disadvantage of this technique is that organoids are not individually addressable for screening purposes. However, it holds promise for fundamental neurodevelopment and disease modelling studies [76]. In a follow-up paper [77], the same authors demonstrated the applicability of this platform, by assessing the impact of nicotine in prenatal brain development. It was found that nicotine exposure elicited neuronal dysfunction, with the organoids presenting impaired neurogenesis, specifically brain regionalization and cortical development [77].

For in vitro studies of neurovascular pathology, Cho et al. [78] designed a 3D BBB model consisting of an assembly of horizontal parallel microchannels beside a tube-shaped macrochannel in a single layered microfluidic chip (Figure 4d). The macrochannel was coated inside with a poly D-lisine (PDL) and a collagen I gel, promoting the adhesion of the RBE4 cell line and leading to the formation after 2–3 days of an endothelial cylindric monolayer that resembles the geometry of small blood brain vessels. The formation of a tight BBB was confirmed by confocal imaging of the positive immunostaining of TJs proteins (ZO-1 and VE-cadherin). Permeability assays with 40 kDa dextran were done in order to test the barrier function. The dye was introduced into the lumen of the macrochannel and the increase in fluorescence on the side channels was monitored. The presence of the BBB slowed the outward flux of the dye, since it took longer to reach gradient saturation in the developed model (7 min), compared to a device without barrier (less than 4 min). Moreover, it was also able to block and inhibit more efficiently neutrophil transmigration upon addition of interleukin 8 (IL-8), a chemoattractant. For probing neuroinflammation response, the BBB was exposed to TNF-α. Elevation of several cytokines (e.g., VEGF, CX3CL1, CINC1, TIMP1, etc.) led to the conclusion that an inflammatory effect was elicited on the BBB model. The platform was also used to study ischemia, induced by oxygen and glucose deprivation followed by reoxygenation. An increase in ROS and Rho-associated protein kinase (ROCK) levels was observed, as well as a decrease by more than half in ZO-1 expression. Antioxidant treatment with edavarone and Y-27632 (a ROCK inhibitor) had limited protective effects in restoring BBB integrity: ZO-1 levels increased slightly after 3h but decreased again after 6h. This can be due to the fact that ischemia is not only a result of oxidative stress, but also hypoxia and other inflammatory factors [78].

## 5. Neurodegenerative Diseases Microfluidic Models

As previously discussed in prior sections, microfluidic devices are able to emulate brain microvasculature-related events, for instance, angiogenesis, inflammation or BBB changes [79]. It is known that BBB dysfunction underlies the development of NDs. By reconstituting such dynamic conditions of the human brain, a validated BBB-on-a-chip can be used to address the critical need to improve the understanding of the pathological mechanisms of NDs [32,79]. Furthermore, since CNS models that mimic NDs typically require controllable fluid delivery and long-term culture duration, microfluidics come forth as an advantageous and reliable platform to study these disorders [39]. A summary of the main findings of the studies that will be discussed in this section can be found in Supplementary information (Table S1).

### 5.1. Alzheimer’s Disease

AD is a neurodegenerative disease that is the most common cause of dementia globally. Pathological hallmarks of AD include the presence of extracellular aggregates of amyloid β (Aβ) plaques and intracellular neurofibrillary tangles of hyperphosphorylated tau protein [80,81]. Anomalous cleavage of the amyloid precursor protein (APP) by β-secretases and γ-secretases leads to the production of insoluble Aβ fibrils, which suffer oligomerization and aggregation into senile plaques. In turn, Aβ concentration triggers the hyperphosphorylation of the tau protein, followed by its polymerization into neurofibrillary tangles. There appears to be a synergistic neurodegenerative effect of these cytotoxic proteins, with the recruitment of microglia cells and formation of a local inflammatory response also potentiating neuronal damage and death [24,81].

AD is associated with three general stages-mild (early), moderate and severe-, with progressive memory deficits and cognitive decline [82]. Neuropsychiatric symptoms may manifest over the course of the disease, including irritability, diminished insight or restlessness. In nearly 30 to 50% of the cases, psychotic symptoms such as hallucinations or delusions of persecution are also present [82]. The plaques and neurofibrillary tangles are mainly detected in cortical and limbic areas of the brain. There is moderate cortical atrophy, accompanied with enlargement of the frontal and temporal horns of the lateral ventricles, and a decrease in brain weight [80,81]. Given that AD is a long-term process, with a lengthy preclinical asymptomatic phase characterized by pathological changes that begin 20 to 30 years prior to the appearance of the first clinical signs, it should not be excluded that cognitively normal individuals might also have the disease [80,82].

Clinical diagnosis entails a thorough assessment of previous medical records and family history to rule out other conditions that may cause similar symptoms, physical and neurological examination, neuroimaging (PET scan, MRI, etc.), and neuropsychological testing, often in combination with detection of biomarkers like Aβ and τ protein in cerebrospinal fluid (CSF) samples from patients [81,82]. To date, accurate identification of AD is still hard, and both clinical diagnosis and post-mortem histopathological examination of the brain are needed in order to give a conclusive diagnosis. This is especially true in the early stages of the illness, primarily because the symptoms are dismissed as a normal result of the aging process. The strongest known risk factor for the development of AD is, in fact, aging, affecting 10% of people over the age of 65 and about 50% of people older than 85. Family history of AD, existence of one or more apolipoprotein E (ApoE) gene alleles, cardiovascular risk factors, and moderate or severe brain traumas can also account for the aetiology of the disease [82].

AD research has been majorly committed to the development of therapies that can reduce aggregates of toxic forms of Aβ and tau protein, with several of them having reached Phase III clinical trials (e.g., NGP 555, verubecestat, immunotherapy vaccines such as aducanumab, etc.) [81,83]. The latest progress is the study by Davtyan et al. [84], wherein they tested in bigenic T5x mice a combinatory therapy that concurrently targeted both Aβ and tau protein, successfully removing plaques and neurofibrillary tangles. Furthermore, the vaccine might also be used as a preventive strategy, since it can likewise decrease the concentration of those proteins in serum and brain extracts, thus averting their deposition [84]. In fact, avoiding plaque accumulation seems to be a better goal, since the removal of plaque deposits might merely result in holes in the neuronal synapses, a damage that can be difficult to overcome. In this field, Novartis has put forth the CAD106 vaccine, presently in Phase II/III study, designed to stimulate the production of Aβ-antibodies [83]. Nonetheless, therapy failure is often frequent, due to unfavourable pharmacokinetics and pharmacodynamics of the drugs [81]. Presently, cholinesterase inhibitors (e.g., donepezil) and memantine, a non-competitive blocker of the NMDA receptor, are available for the treatment [85].

Park et al. [86] were able to show the neuroinflammatory role of microglia in AD pathogenesis, and their influence in neuron death. The investigators developed a 3D human AD triculture system, by culturing neurons and astrocytes expressing mutant APP in a central chamber coated with Matrigel, with human immortalized SV40 microglia being added in surrounding angular chambers at a later time. The neurons and astrocytes expressed late-stage AD hallmarks, including Aβ aggregation, abundant tau protein formation and secretion of pro-inflammatory cytokines and chemokines, thus recapitulating many of the features of this disease in a single microfluidic platform. Compared with controls, in this model, infiltrating microglia migrated quicker and induced greater neuron toxicity/death by a pathway involving TLR 4 and interferon-γ (IFN-γ). This was demonstrated by the fact that using IFN-γ and TLR4 neutralizing antibodies led to a reduction in microglial activation and, consequently, neuronal toxicity [86].

Concave microwell arrays in a microfluidic system were used to seed and culture for up to 10 days neural progenitor rat cortical cells, with or without fluid flow, evaluating thereafter the neurotoxic effects of Aβ on the formed 3D gel-free neurospheroids [87]. The group demonstrated that, compared to static cultures, the neurospheroids grown under perfusion conditions similar to those of the CSF in the brain, had generally larger sizes and greater neurite extension, resulting in a more complex and robust neural network. Furthermore, cell staining for β-III tubulin marker, denoted an enhanced differentiation of the progenitor cells into mature neurons. These differences could be the result of a better access to oxygen and nutrients, and continuous clearance of metabolic wastes provided by the fluid flow. Afterwards, the neurospheroids were exposed to Aβ, in the same experimental settings. The results showed that Aβ was significantly more toxic to neurospheroids under flow conditions induced by an osmotic pump, causing greater destruction of neural networks and significantly decreasing cellular viability [87].

**Figure 5:**
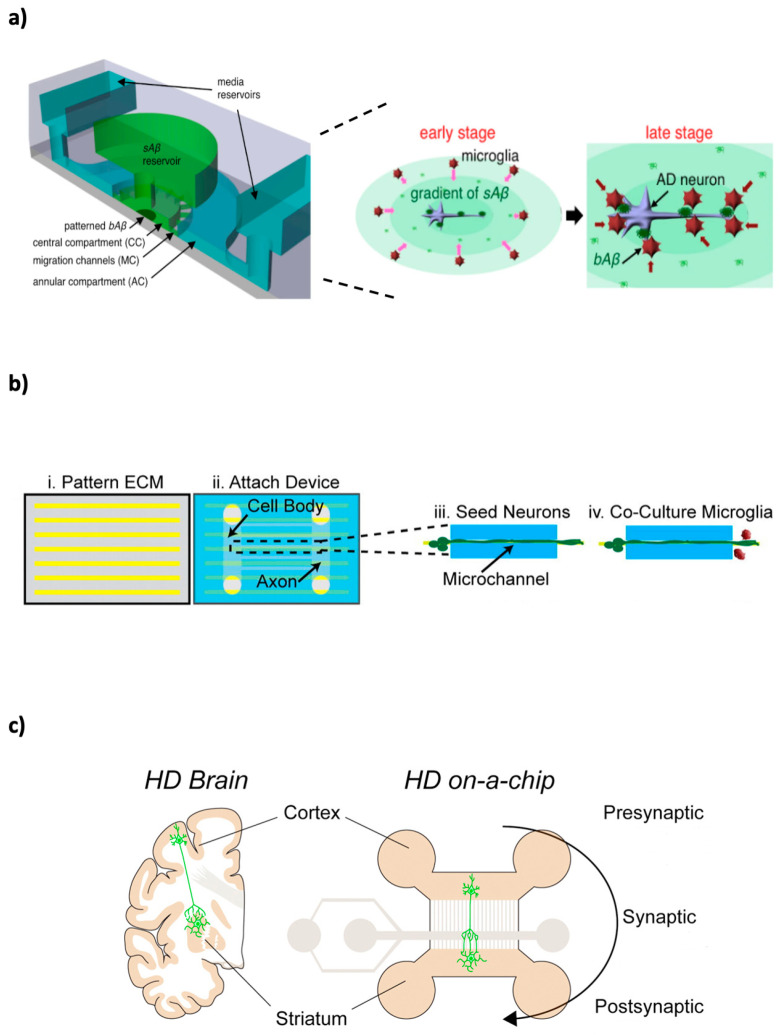
Schematic representation of some microfluidic models for neurodegenerative diseases, namely: (**a**) Alzheimer’s disease, (**b**) Multiple sclerosis, (**c**) Huntington’s disease. Reprinted and adapted with permission from: (**a**) Cho et al. [88], (**b**) Hosmane et al. [105], (**c**) Virlogeux et al. [113].

Cho et al. [88] elucidated the role of Aβ on microglial accumulation. They cultured human microglial cells in a microfluidic platform and studied their response to chemotaxis of week-long gradients of soluble Aβ and surface-bound (insoluble) Aβ (Figure 5a). Time-lapse microscopy showed that soluble Aβ drives microglia migration towards the central chamber, explained by Aβ-induced secretion of the chemoattractant MCP-1. Furthermore, microglial motility was decreased in areas with Aβ fibril-coated surfaces, which correlates to what is seen in vivo in AD brains, where microglia cells form a stable association with Aβ deposits. All in all, there seems to be a synergistic effect between soluble and insoluble Aβ on microglial recruitment and localization during the neurodegenerative process of AD [88].

Song et al. [89], to the best of our knowledge, were the first group to deliver direct experimental evidence of extracellular Aβ spreading through neuronal connections. They demonstrated retrograde transport of Aβ from axons to the neuronal cell bodies, being subsequently secreted and transmitted to neighbouring neurons [89]. Deleglise et al. [90], examined the impact of Aβ on neuron degeneration patterns. By using a microfluidic chip comprised of two chambers separated by microchannels, the scientists were able to culture primary cortical and hippocampal mouse neuronal cells and isolate soma-dendrites from axonal projections, applying afterwards Aβ peptides to the different cellular compartments. It was found that local somatodendritic Aβ deposits trigger a rapid distal presynaptic loss and disconnection, long before soma/dendrite abnormalities and death start to occur (a phenomenon referred to as “dying-back process”). On the contrary, when the axons where treated with Aβ, the same was not observed. This demonstrates local Aβ deposits are efficient in generating signalling pathways (e.g., caspase and NAD^+^ pathways) that lead to early degeneration in anatomically distant areas of the neuron [90]. To this day, there are doubts about which molecular entity of Aβ is the most toxic and responsible for causing AD. Choi et al. [91] have looked into this matter. They exposed neurons (differentiated from rat neuronal progenitor cells) adhered onto a microchannel of a PDMS microchip to a slow gradient of Aβ oligomeric assemblies for 3 days. Although fibrilization increased considerably over time, cell viability did not statistically change. This suggests that Aβ fibrils do not have a significant role in neurotoxicity, but rather oligomeric assemblies potentially contribute to the neurodegeneration in AD [91].

To determine the nature of the tau responsible for neuron-to-neuron transmission, Takeda et al. [92] developed a compartmentalized 2D microfluidic platform comprised of three interconnected chambers wherein two sets of neurons where cultured. The authors collected different tau species from cortical extracts and interstitial fluid of human and transgenic mice brains, and their propagation through the synaptic connections was observed over the course of 14 days. A soluble high molecular weight phosphorylated tau was identified as the bioactive form, undergoing direct trans-synaptic transport and initiating the seeding that leads to the formation of aggregates within the cytoplasm of the neurons [92]. Wu et al. [93] further demonstrated in a study using a tripartite microfluidic device that cell to cell propagation is capable of inducing tau aggregation in downstream neurons [93].

### 5.2. Parkinson’s Disease

First described in 1817 by James Parkinson, PD is after AD the second most prevalent neurodegenerative disease [94]. Estimations point towards 1-3% of the population over the age of 65 developing this illness, with people under 40 years rarely being affected [95]. PD involves progressive disruption and loss of dopaminergic neurons in the substantia nigra pars compacta, with substantial depletion of dopamine [94]. Neuronal loss is also extensive in cholinergic (basal nucleus of Meynert), serotonergic (raphe nuclei) and noradrenergic (locus coeruleus nuclei) systems, in the brainstem (dorsal motor nucleus of the vagus nerve and pedunculopontine nucleus), in the cerebral cortex, optic bulb and autonomic nervous systems [95,96]. Accumulation of cytoplasmic inclusions consisting of α-synuclein (αSyn) aggregates, called Lewy bodies, is likewise present in the affected areas. It is not known how Lewy bodies specifically relate to the progression of the disorder, but nevertheless, the formation of these aggregates induces neurodegeneration [94,95]. Since the disease is heterogeneous and multifactorial, protein aggregations are not the only underlying reason for neuronal loss. Multiple other cellular processes, such as mitochondrial dysfunctions, neuroinflammation, oxidative stress, impaired bioenergetics, altered activity of calcium channels or genetic mutations, equally contribute to PD development of PD [95].

PD manifests itself through motor and nonmotor symptoms. Dysfunctions of the somatomotor system comprise resting tremor (usually unilateral, in the beginning), bradykinesia (slowness of voluntary movements), impaired gait, postural instability and rigidity. The diagnosis is usually made after the onset of one or more of these core motor features [95,96]. Throughout the course of the disease, nonmotor symptoms also arise: cognitive decline, mood and sleep disorders, and dysfunction of the autonomic nervous system, including constipation, orthostatic hypotension, changes in thermoregulation, incontinence or erectile impotence. These can be as debilitating as the motor symptoms, increasing disability and reducing quality of life. Some of the key signs are a direct result of neurodegeneration, while other clinical features are suspected to be caused by aberrant activity patterns within surviving neurons [94,96]. PD is still incurable, but management of the disease through replacement therapy with dopamine precursor levodopa or dopamine agonists, can be helpful in symptomatic relief [95,96]. Additional options include dopamine agonists, catechol-O-methyltransferase (COMT) inhibitors, anticholinergics and monoamine oxidase-B (MAO-B) inhibitors [97].

Moreno et al. [98] were successful in differentiating human neuroepithelial stem cells embedded in Matrigel into dopaminergic neurons, using a 3D microfluidic phase-guided bioreactor. Immunofluorescence staining showed a differentiation efficiency of around 19%, which was comparable to that of a macroscopic culture. The biocompatibility and biological fidelity of this new model were further confirmed by the electrophysiological activity of the dopaminergic neurons. This model could be an economically efficient route to personalized drug discovery for PD [98]. Kane et al. [99] established the first fully automated 3D brain-on-a-chip (termed “Pelican”) for microfluidic cell culture and real-time monitorization through image acquisition. Using the Pelican, they differentiated PD patient stem cells into midbrain dopaminergic neurons. Long-term maintenance was achieved (over 100 days) and on day 24, quality control of the culture was assessed. Immunostaining revealed the presence of fully mature neurons within the microchips and calcium imaging confirmed their electrophysiological activity. The authors denote that this is a flexible cost-effective approach to not only model PD, but also for other diseases, enabling adaptation to various experimental protocols. Integration of new modules could expand even more the array of possible in vitro tests [99]. In a second study, Bolognin et al. [100] also resorted to patient stem cells to generate a 3D microfluidic model with neurons carrying the LRRK2-G2019S mutation (responsible for autosomal-dominant PD). The mutation led to progressive dopaminergic degeneration with mitochondrial defects. When compared to a 2D system, the 3D culture presented more robust PD endophenotypes, thereby demonstrating a higher degree of differentiation into the intended specific subtype. Treatment with LRRK2 inhibitor 2 was effective in lessening neurodegeneration and in rescuing some dopaminergic phenotypes, indicating that this platform may be useful for drug screening purposes [100].

Fernandes et al. [101] explored the molecular mechanisms involved in PD using a microfluidic chip. The device consisted of two culture chambers interconnected by three channels. Integrated pneumatic valves allowed fluid flow control and communication between the two chambers. In the first experiment, the transmission of αSyn between two cell populations (naïve human H4 neuroglioma cells and H4 cells expressing αSyn tagged with GFP fluorescent marker) was assessed. They permeabilized the membrane of the disease-model cells to release αSyn to the medium, monitoring thereafter the protein diffusion to the other chamber of the device, where the healthy naïve H4 cells were present. There was no detectable uptake of αSyn-GFP on the part of naïve H4 cells. Several possible explanations might justify this result, such as: too low concentration of αSyn-GFP being released, rapid degradation of the molecule or even the high molecular weight of GFP affecting the spreading of αSyn. Nevertheless, the authors point out that the platform is still valid to study cell–cell communication via soluble factors. The second experiment entailed evaluating the impact of activated N9 microglia cells on the neuron-like H4 population. H4 cells co-cultured with the LPS-activated N9 cells presented almost 2-fold higher ROS levels than the controls, confirming the interplay and crosstalk between the two cell lines [101].

Freundt et al. [102] seeded and cultured primary neurons for one week in a microfluidic device, in the presence of αSyn fibrils. Using live cell imaging and immunofluorescence, the group showed that the fibrils are internalized and transported anterogradely along the axons until reaching the neural soma, following which, they are released and transferred to other neurons. This mechanism can help explain the characteristic pattern of neuron-to-neuron spread of Lewy bodies between connected brain areas, such as the neocortex and the limbic system [102].

Mitochondrial trafficking defects are implied in PD pathogenesis. Bearing this in mind, Lu et al. [103] created a compartmentalized microdevice that allowed visualization of mitochondria transport in dopaminergic axons. Upon application of the PD-mimetic toxin MPP^+^, a rapid (<1 h) and selective decrease of mitochondrial movement was observed [103].

### 5.3. Multiple Sclerosis

MS is an autoimmune inflammatory neurodegenerative disorder, characterized by nerve demyelination and axon damage. Myelin proteins start to get recognized as foreign components by the immune system, resulting in the destruction of the myelin sheath [97]. Apoptosis of oligodendrocytes, the cells responsible for forming and maintaining myelin, instigates further neuronal breakdown and loss of function. Over a dozen of drugs with different mechanisms of action have been approved by the Food and Drug Administration (FDA) to treat MS attacks (e.g., dimethyl fumarate, interferon-β (IFN-β), fingolimid, teriflunomide, ocrelizumab, etc.) [97].

Given the importance of the possibility of remyelination in MS, Kerman et al. [104] combined stem cell biology and microfluidic technology to differentiate mouse embryonic stem cells into myelinating oligodendrocytes, subsequently assessing and quantifying myelin formation over several days. Live imaging and confocal analysis led to the observation that oligodendrocytes anchor to the bare axons before wrapping them and forming the myelin sheets. This model is a suitable tool to better comprehend the myelination process and to unravel novel treatments for demyelinating diseases like MS [104].

Hosmane et al. [105] created a co-culture microfluidic system to study axons-microglia interactions (Figure 5b). The group induced axon degeneration and found that there is microglial clearance and phagocytosis of unmyelinated axonal debris through toll/interleukin-1 receptor domain-containing adapter inducing interferon-β (TRIF)—a Toll-like receptor adapter protein—, and through p38 mitogen-activated protein kinase (MAPK), thus seemingly showing the importance of these cells in contributing to neural repair after peripheral insult [105].

### 5.4. Amyotrophic Lateral Sclerosis

In ALS, a motor neuronal disease, there is progressive degeneration of upper and lower motor neurons in the corticospinal tract. In consequence of the deterioration, communication between the motor neuron and the muscle is lost. ALS usually begins in the 40s or 50s with painless, localized weakness of the arms and legs, gradually evolving to affect most muscles, with patients dying within 3 to 5 years of the initial diagnosis. Although the exact mechanism is still unclear, it is thought that several processes contribute to the development of ALS, including genetic factors, glutamate excitotoxicity, inflammation, mitochondrial and enzymatic dysfunction or ROS [32,97]. Two forms of the illness are known: sporadic ALS, the most prevalent, comprising around 90% of the cases, and familial ALS, where there is a family history of the disease relating to a genetic component. Until now, no effective treatment was found to cure ALS. Only two drugs approved by FDA, riluzole and edavarone, provide a very limited enhancement in survival [32,97].

Recently, several microfluidic models have allowed to better understand neuromuscular junction (NMJ) connectivity and pathophysiology. For instance, Machado et al. [106] developed a system that is designed in such a way that motor neurons in an outer compartment are able to extend their axons through an array of microchannels and form NMJ with myofibres seeded in the central compartment. The validation of key NMJ features, such as axon outgrowth and proper myofibre contraction, were verified by immunostaining with β3-tubulin antibody and optogenetic stimulation, respectively. By co-culturing the motor neurons derived from mouse embryonic stem cells with astrocytes expressing superoxide dismutase enzyme 1 (SOD1) (an ALS-linked mutation), and with myofibers, the authors observed denervation and diminished contractile response, central features of this disorder. Furthermore, they verified that through applying the RIPK1 inhibitor Necrostatin, an ALS drug candidate, the phenotype could be reversed, thus improving motor neuron survival and reducing the deterioration of motor innervation [106]. Southam et al. [107] demonstrated the importance of glia cells in motor neuron growth and spreading in a physiologically relevant NMJ microfluidic model [107]. In a 2013 study, Ionescu et al. [108] also established a microdevice that enabled the efficient monitoring of neuron-muscle formation, maintenance and communication through imaging, calcium transient recording and muscle contraction assay [108].

Evidence suggests that non-cell autonomous processes have a role in ALS neurodegeneration, with neighbouring glial cells such as astrocytes secreting proinflammatory cytokines and inducing further neuronal death [97]. To better understand these indirect interactions and extracellular metabolic communication, Kunze et al. [109] co-cultured neurons with astrocytes that overexpress either a wild-type (WT) or mutated SOD1, assessing afterwards the response in terms of neuronal cell activity. They showed that the cortical neurons in metabolic contact with SOD-mutant astrocytes had a reduction in cell density of about 45%, as well as loss in synapsin protein expression. In contrast, SOD-WT overexpressing astrocytes reduced oxidative stress on cortical neurons that were in close metabolic contact [109].

Spinal motor neurons are considered to be highly vulnerable in ALS. Sances et al. [110] showed that co-culturing brain microvascular endothelial cells with motor neurons, both derived from iPSC, resulted in vascular-neural interaction and activation of specific spinal cord developmental genes, considerably enhancing in-vivo like spinal neural tissue maturation, function and signalling. This model could help unravel the mechanisms behind motor neuron-related diseases, provide a platform to infer the penetrance of prospective ALS therapeutics into the blood-spinal cord barrier and study the modulation of neural activity [110].

Osaki et al. [111] created a human ALS microfluidic model, by co-culturing motor neurons spheroids derived from iPSC of a patient with sporadic ALS with 3D skeletal muscle fibres surrounded by an endothelial cell layer, in a collagen gel. Compared to a normal motor neuron model (control), the ALS system had motor neuron degeneration, increased muscle apoptosis and atrophy, and reduced contraction force. To study potential drugs that might confer neuroprotection, both the control model and the ALS model were treated with rapamycin (an mTOR inhibitor) and bosotunib. With the tested therapeutic agents, the disease model exhibited less neurotoxicity, superior motor neuron survival and improved muscle contraction. This was attributed to an induction and up-regulation of autophagic processes, and to the degradation of TAR DNA binding protein 43 in the motor neurons. Although the endothelial cell barrier is known to hinder drug penetration, rapamycin and bosotunib co-treatment reduced the expression of P-gp efflux pump, significantly increasing muscle contraction force. Furthermore, to mimic glutamate excitotoxicity, the control culture was exposed to an excess of glutamic acid for 14 days. The results showed that it caused motor neuron dysfunction and death, along with neurite regression and muscle atrophy. Given that other NDs such as AD or PD have also been associated with muscle strength, the authors suggest that the developed microplatform could be a robust model to facilitate the investigation of neurovascular coupling in those cases as well [111].

Tan et al. [112] studied the neurotoxicity of β-methylamino-L-alanine (BMAA), a cyanotoxin that has been linked with neurodegeneration in ALS. Using a microfluidic device, the group treated mature rodent cortical neurons with BMAA, observing axonal degeneration at sublethal concentrations, as well as rapid BMAA transcellular forward spreading to other neurons, which could possibly be associated with ALS progression [112].

### 5.5. Huntington’s Disease

HD is an illness with autosomal dominant inheritance caused by an expansion of CAG trinucleotide repeats in the gene encoding the protein huntingtin, resulting in the generation of a mutant huntingtin (mHTT). mHTT induces dysregulated gene expression, formation of aberrant toxic inclusion bodies, impaired protein folding and degradation, mitochondrial dysfunction, among other things, all of which lead to neuronal degeneration and death [32,97]. The earliest symptoms are rapid and involuntary movements that affect mostly facial muscles and distal limbs, termed “chorea”. Currently, no approved therapy can delay the advance of HD. However, tetrabenazine is frequently used to supress the chorea [97].

Virlogeaux et al. [113] reconstituted a corticostriatal network in a microdevice, demonstrating that presynaptic defects contribute more to the progression of HD than previously thought. Indeed, HD cortical neurons expressing mHTT caused functional and signalling impairments in striatal neurons (e.g., compromised glutamate release), altering the global integrity of the whole network. On the other hand, WT cortical neurons were sufficient to rescue and restore the circuit in HD striatum, improving survival signalling. It can be concluded therefore that the genetic status of presynaptic neurons plays a crucial role in HD striatum dysfunction and neurodegeneration. Some of the benefits of this system remarked by the authors are its suitability for spatial and high temporal resolution imaging, testing drugs for the treatment of HD or getting further insights into pathophysiological mechanisms [113].

Vatine et al. [114] used patient diseased iPSC lines to create an HD-on-a-chip and investigate the BBB dysfunction in this disease (Figure 5c). Permeability for dextran of several molecular sizes was increased, suggesting a significant disruption of the integrity of the vascular barrier. The model may thus be used for several aspects of disease modelling, including predict interindividual variability in BBB function and in CNS drug penetrability [114].

## 6. Microfluidic Synthesis of CNS-Targeted Nano/Microcarriers and Other Compounds

Microfluidics production of NPs is a relatively established research field. However, when it comes specifically to the microfluidic synthesis of nano-/microcarriers or other compounds (e.g., radiotracers) meant to target the CNS, either with therapeutic or diagnostic purposes, to the best of our knowledge, only a few studies have been published, which we will proceed to describe in this section.

**Figure 6:**
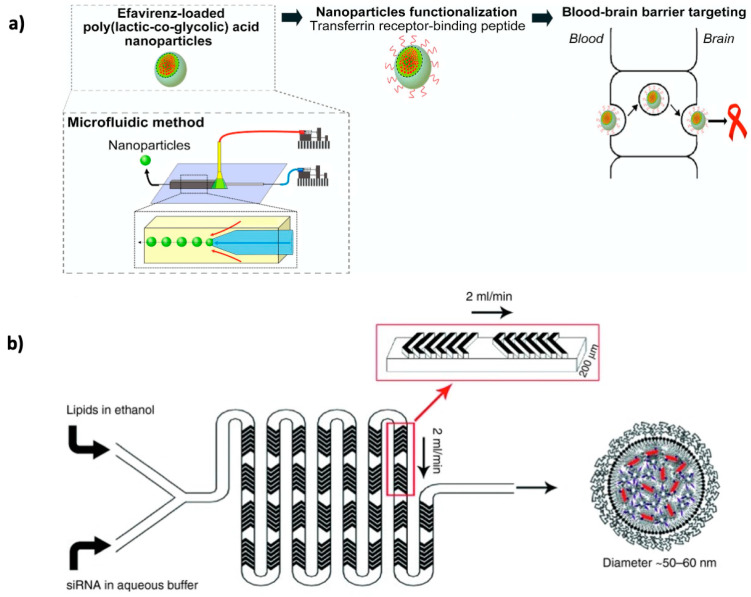
Production of CNS-targeted nanocarriers through microfluidic devices: (**a**) Tranferrin functionalized poly(lactic-co-glycolic) acid (PLGA) nanoparticles (NPs) for the treatment of HIV neuropathology, (**b**) siRNA-loaded lipid NPs produced with a staggered herringbone microfluidic mixer, intended to silence neuronal gene expression. Reprinted with permission from: (**a**) Martins et al. [19], (**b**) Rungta et al. [119].

The human immunodeficiency virus (HIV) is able to infect the brain and use it as a reservoir for replication. Bearing this in mind, Martins et al. [19] prepared transferrin functionalized poly(lactic-co-glycolic) acid (PLGA) NPs, loaded with efavirenz (an anti-HIV drug), in order to target the BBB and treat HIV neuropathology (Figure 6a). Relative to a conventional nanoprecipitation technique, the microfluidic approach produced particles with a smaller size (83 nm versus 133 nm), higher drug loading (10.8% versus 3.2%) and association efficiency (80.7% versus 32.7%). A sustained in vitro release was observed, with the nanosystems releasing 50% of the drug within 24h. Although the functionalized NPs had a 1.3-fold higher permeability than the free drug across a hCMEC/D3 in vitro model, showing their capability to interact more efficiently with BBB cells and leading to a greater anti-HIV effect, in regard to plain PLGA NPs, the transferrin formulation did not potentiate efavirenz membrane permeation. Furthermore, the NPs were found to be non-haemolytic and non-toxic to BBB endothelial and neuron cells (metabolic activity above 70%), being therefore safe for the intended intravenous administration and opening new doors for advancements in HIV neuropathology [19].

Precise imaging of cerebrovascular structures can significantly contribute to the timely diagnostic and treatment of NDs, including brain tumours. Several modalities are available, such as near-infrared II (NIR-II) fluorescence or photoacoustic imaging. For this purpose, Guo et al. [115] synthesized a theranostic nanosystem, by producing conjugated polymer NPs with a uniform size of 50 nm, high imaging contrast and excellent photostability. The NPs were successfully used as contrasting agents in dual NIR-II fluorescence and photoacoustic imaging, to efficiently and noninvasively map deep microscopic brain tumours in nude mice after focused ultrasound (FUS) induced BBB opening. The results from cytotoxicity assays in bEnd.3 and C6 cells (> 95% cell viability) and the fact that no inflammatory lesions or tissue damage were found in organs collected from the treated mice, demonstrated that the nanocarriers are highly biocompatible [115]. In another study performed by this group in 2019 [116], it was possible to obtain high-resolution 3D images of the cerebral cortex vasculature, ear blood vessels and angiogenic tumour vessels of mice, using the same techniques and nanosystem. By optimizing the microfluidic conditions (320 of Reynolds number for fluid flow and 40% ethanol volume fraction), they obtained NPs with the smallest average diameter of 40 nm [116]. The authors envision that their polymer NPs can improve diagnostic accuracy of brain tumours and other neurodegenerative processes, as well as enhance real-time imaging guided neurosurgical and photothermal treatment [115,116].

Jgamadze et al. [117] developed poly(N-isopropylacrylamide) (pNIPAM) hydrogel particles to facilitate neuronal cell growth and transplantation into the hippocampus of young adult rats. Modification with polylysine coating led to the immobilization and adhesion of progenitor neuronal cells on the external surface of the microcarriers, making expansion and differentiation into neurons successful. Upon injection of the composites into the target site, robust cell implantation was observed. Compared to conventional borosilicate glass beads, the pNIPAM microcarriers provided an increase of 2.7-fold in the number of implanted cells. This was attributed to the rapid thermoswitching, stimuli-responsive nature of the pNIPAM polymer, which promoted the release of the mature neurons without damaging the neuronal processes, thus contributing to their successful long-term survival and integration for at least 24 weeks in the rats. Furthermore, contrary to what is sometimes observed with glass beads, no cumpling of pNIPAM particles was noted at the injection site, which indicates low risk of long-term inflammatory responses. It can be concluded that the properties of pNIPAM microcarriers make them a promising and efficient alternative to direct cell transplantation [117].

MicroRNA interference (miRNA) therapy holds great potential to treat many disorders, including NDs. Samaridou et al. [118] engineered RNA-loaded cationic nanocomplexes for direct nose-to-brain delivery. The formation of the nanocomplexes was based on electrostatic interactions between a modified octaarginine (C12-r8) and the RNA molecules. Two different polymers, hyaluronic acid (HA) and polyethyleneglycol-polyglutamic acid (PEG-PGA), were used to envelop the nanocomplexes, in order to protect against enzymatic degradation and enhance diffusion across the nasal mucosa. Tweaking the microfluidic conditions (i.e., increasing the concentration or the flow rate ratio of C12-r8:RNA), enabled the rapid manufacturing of a scalable nanosystem with a reproducible mean size of 70 nm and an association efficiency close to 100%. The size of 70 nm is important, since it is known that NPs with or less than 100 nm can enable a better transport across the BBB. The nanocarriers were able to preserve their colloidal stability in biorelevant conditions, preventing the premature release of the RNA. Moreover, in vitro assays revealed that the HA and PEG-PGA nanocomplexes were very efficient in interacting and being internalized by CHO cells, given that a markedly higher cellular uptake was observed when compared with a free miRNA mimic. Following intranasal administration in an AD mouse model, the NPs successfully increased the therapeutic miRNA in the hippocampus area. This, in turn, led to a decrease in the expression levels of GATA2 and Rb1 proteins, which play a role in cell cycle and are upregulated in AD. Overall, the findings suggest that the RNA nanocarriers developed by Samaridou et al. are a promising therapeutic strategy for AD [118].

Rungta et al. [119] reported siRNA-loaded lipid NPs effective in silencing neuronal gene expression, both in vitro and in vivo, after intracranial injection. The NPs (lipid composition of 3-(dimethylamino)propyl(12Z,15Z)-3-[(9Z,12Z)-octadeca-9,12-dien-1-yl]henicosa-12,15-dienoate (DMAP-BLP), distearoylphosphatidylcholine (DSPC), cholesterol and PEG-DMG) were produced using a staggered herringbone microfluidic mixer (Figure 6b). The authors demonstrated that the nanocarriers accumulate inside neurons in culture in an ApoE-dependent manner, resulting in a 100% efficacy uptake. Delivery of siRNA lipid NPs to the brain of rats was successful, leading to widespread knockdown of neuronal genes that encode for the PTEN and GRIN1 proteins, with a significant reduction of 72% and 51% in expression levels, respectively, compared to noninjected controls. Furthermore, no significant immunogenicity or toxicity was detected, which is indicative of their biocompatibility and improved safety. The results show therefore that the siRNA lipid formulation was capable of manipulating the expression of molecular targets involved in neuronal processes, potentially facilitating the development of gene therapies for NDs [119].

Yu et al. [120] employed lipopolymeric NPs (LPNPs) to minimize side effects and enhance therapeutic outcomes in glioblastoma. The LPNPs encapsulated different siRNA to target four transcription factors (OLIG2, POU3F2, SOX2 and SALL2) that lead to the formation of brain tumour-initiating cells, which in turn drive recurrence, malignant growth and therapy resistance. Different ratios of C15 epoxide-terminated lipids, DSPE-epoxy-PEG 2000 and polyethyleneimine 600 were used to obtain LPNPs with a size span from 40 to 135 nm. Infusion of a high dose of LPNPs (1.5 mg/kg), achieved by intratumoral injection and convection enhanced delivery, resulted in an extension of median survival of 19 days in patient-derived xenograft glioblastoma mouse models (50% increased survival). In contrast, while limiting the dose attenuated tumorigenesis, it did not offer any survival benefit. This study suggests that multiplexed nanosystems can be a viable option to overcome the challenges posed by tumour heterogeneity [120].

Radiotracers are chemical compounds used for positron emission tomography (PET), a non-invasive medical imaging method that can be employed for several purposes, such as to diagnose and determine disease stage, evaluate treatment efficacy or research the biological processes involved in pathology development, including NDs. Recently, Zhang et al. [121] reported a simple PDMS microfluidic system for the radiosynthesis of [^18^F]fallypride. The highly integrated configuration of the platform allowed to perform in a short time (~60 min.) most of the necessary reaction processes, including [^18^F]fallypride concentration and purification, with good radiochemical yield (~87%) and purity (~99%) of the tracer. It was possible to obtain a sufficient quantity of [^18^F]fallypride for multiple rat injections for preclinical brain imaging studies, including of the cerebellum, left striatum and right striatum. Despite such promising results, improvements are still needed in regards to scale-up, in order for microfluidics to be widely adopted as a viable alternative to traditional radiotracer production methods [121].

## 7. Challenges, Future Perspectives and Conclusions

Microfluidics has been around for 30 years, having undergone significant advances since its inception in the early 1990s. However, it has yet to achieve its full potential within the fields of chemical and biological experimentation [9,46]. There are several practical problems that need to be overcome if these systems are to be used at a wider, commercial scale in the applications that have been discussed in the prior sections [46].

When it comes to organs-on-a-chip, functionality is currently limited. Perfusion, cell injection and sampling have a low throughput character, despite the existence of automation hardware. The initial preparation of the device and injection of cells are often still a very manual process. Likewise, assurance of sterile handling is critical for proper cell culture and long-term maintenance. Such procedures can be tricky and cumbersome, if not done by a trained operator [122]. Many times, the real usefulness of these models, as is the case of BBB/brain-on-a-chip, is translated in the fact that they allow for simultaneous monitoring and recording of biological responses to a number of stimuli [123]. However, on-chip sample collecting is not very practical nor feasible, since it can interfere with its operation, risking changes in the concentration of biomarkers/metabolites and potentially generating data that cannot be correlated with what happens in vivo [124]. The integration of more sensible and cost-effective sensors that perform the analysis independently in the micro-space of the chip is therefore required [122,123]. Appropriate endpoints for readouts also need to be established [122]. Progresses in each of these areas will allow the creation of more high-end, sophisticated, but still, nonetheless, practical platforms, that can facilitate the early diagnosis of various NDs and the development of effective therapeutic agents [6].

Another relevant point is the source of the biological tissue [124]. Induced pluripotent stem cells (iPSC) are a very attractive choice for cell culture in microfluidic systems, given their virtually unlimited ability to self-renewal and differentiate into multiple somatic lineages [7,41]. Indeed, extensive work has already been conducted in regard to the incorporation of stem cells in BBB/brain-on-a-chip for engineering neural networks and brain tissues [41]. iPSC circumvent the ethical controversies associated with traditional embryonic stem cells and maintain several BBB physiological attributes, including the presence of TJ proteins and similar molecular permeability coefficients [7,124]. Furthermore, they can be obtained from individual patients, which will drive forth not only the development of patient-specific brain disease models, but also of personalized drug screening. Accordingly, and despite several limitations on differentiation and maturation protocols that still subsist, iPSC is likely to become the main cell source for organs-on-a-chip [7,124].

Regarding the production of NPs, microfluidics could offer an avenue to meet industrial scale-up benchmarks, by means of parallelization, automation, employment of continuous flow and faster mixing rates. This would result in an increase of the total output, reduce batch variability, and diminish human error, potentially allowing the marketing of less expensive nanomedicines to a greater patient population [18]. Notwithstanding the abundance of proof-of-concept publications, there are currently no commercial nanopharmaceutics manufactured using microfluidics. Several reasons might explain the lack of successful translation [18]. Firstly, the cost associated with mass production and experimental implementation of microfluidic setups is very high. Similarly, there is an absence of standard protocols for large-scale production of the devices. Thus, it is imperative to come up with robust and transversal manufacturing protocols that facilitate bulk fabrication of cheap, reusable and easy to dispose components [46,124]. Secondly, the conservatism and reluctance of the pharmaceutical industry. This is a business that owing to its strict regulatory requirements, relies heavily on traditional manufacturing approaches. There would have to be a substantial cost reduction or operational advantage to encourage the use of microfluidic technology beyond the laboratory scale [18]. In this context, direct collaboration between companies and researchers from different fields should be fostered, in order to truly understand the needs of the industry and adapt the designing processes accordingly [46]. Adopting 3D printing as the main strategy for producing microfluidic chips could also help in this regard, since this technique is low-cost, easy to use, efficient and allows for rapid prototyping and quick translation of new designs [18]. Lastly, broad acceptance by the general public is a major challenge. Costumers would need to be reassured that quality control standards would still be met, and that the final pharmaceutical product was produced in compliance with regulatory practices [18]. Nonetheless, the use of microfluidics is paving the way to revolutionize the landscape of pharmaceutics, and it is envisioned that with continued progress, it will be possible to bridge the gap between the lab and the clinic [8].

## Supplementary Materials

The following are available online at https://www.mdpi.com/1999-4923/12/6/542/s1, Table S1: Summary of the main findings of the studies on neurodegenerative diseases using microfluidic devices.
