# Supplementary material for: Recent Developments in Microfluidic Technologies for Central Nervous System Targeted Studies
Source: Pharmaceutics. 2020 Jun 11;12(6):542. doi: 10.3390/pharmaceutics12060542 (PMC7356280; doi:10.3390/pharmaceutics12060542)
Supplement: Supplementary file 1 [file pharmaceutics-12-00542-s001.pdf]

Supplementary Materials

Table S1. Summary of the main findings of the studies on neurodegenerative diseases using microfluidic devices.

Disease	Device Design	Cells	Findings/Results	Reference
Alzheimer's disease (AD)	3D PDMS device with two chambers	Mutant APP-expressing human neurons and astrocytes, human SV40 microglia	1. Aβ aggregation, abundant tau protein formation and secretion of pro-inflammatory cytokines 2. Quicker migration and greater neuron toxicity/death induced by microglia in this model, compared with controls	[86]
	PDMS system containing concave microwells	Rat neural progenitor cortical cells	1. Neurospheroids grown under perfusion conditions had larger sizes, greater neurite extension and enhanced differentiation, compared with static culture 2. Aβ was significantly more toxic for the neurospheroids under flow conditions, causing more destruction of neural networks and significantly decreasing cellular viability	[87]
	PDMS chemotaxis microplatform	Human microglia cells	1. Soluble Aβ has a chemotatic effect, leading to microglial recruitment and migration 2. Insoluble Aβ reduces microglial mobility and viability	[88]
	PDMS microfluidic system	Rat cortical neuron cells	1. Aβ is transmitted extracellularly via neuronal connections of neighbouring neurons	[89]
	Microfluidic chip with two chambers separated by microchannels	Mouse primary cortical and hippocampal neuron cells	1. Aβ deposits trigger presynaptic loss and disconnection, long before soma/dendrite abnormalities and death start to occur ("dying-back process")	[90]
	PDMS microfluidic system	Rat neuronal progenitor cells	1. Cell viability of neurons exposed to a gradient of Aβ oligomeric assemblies for 3 days did not statistically change, suggesting that Aβ fibrils do not have a significant role in neurotoxicity	[91]
	2D microfluidic system with three interconnected chambers	Mouse primary cortical neuron cells	1. Identification of the tau species responsible for neuron-to-neuron propagation (soluble high molecular weight phosphorylated tau)	[92]

			2. The bioactive form undergoes direct trans-synaptic transport and initiates the seeding that leads to the formation of aggregates within the cytoplasm of the neurons	
	Tripartite microfluidic chamber device	Mouse primary cortical and hippocampal neuron cells	1. Cell to cell propagation is capable of inducing tau aggregation in downstream neurons	[93]
Parkinson's disease (PD)	3D microfluidic phase-guided bioreactor	Human neuroepithelial stem cells	1. Successful differentiation of neuroepithelial stem cells into dopaminergic neurons (around 19%), comparable to that of a macroscopic culture<br>2. Biocompatibility and biological fidelity of the model further confirmed by the electrophysiological activity of the dopaminergic neurons	[98]
	Stratified array of 96 microfluidic chips embedded in a customized 384-well microtiter plate format (Organoplate®)	Human PD neuroepithelial stem cells	1. Successful differentiation of PD patient neuroepithelial stem cells into midbrain dopaminergic neurons.<br>2. Long-term maintenance (over 100 days) of fully mature neurons with electrophysiological activity within the microchips	[99]
	Organoplate®	PD human neuroepithelial stem cell lines (carrying the LRRK2-G2019S mutation)	1. Dopaminergic neurons LRRK2-G2019S mutation led to progressive dopaminergic degeneration with mitochondrial defects.<br>2. Compared with a 2D system, the 3D culture presented more robust PD endophenotypes, demonstrating a higher degree of differentiation into the intended specific subtype.	[100]
	PDMS system containing two culture chambers interconnected by three channels	Human H4 neuroglioma cells and N9 microglia cells	1. Increase in the levels of ROS in H4 cells cultured in the presence of activated N9 cells, confirming the cross talk between different cell populations<br>2. Validation of the platform to study cell-to-cell communication and the molecular mechanisms of PD	[101]
	PDMS device with four chambers and two channels	Mouse primary cortical neurons	1. α-synuclein aggregates are internalized and transported anterogradely along the axons, being released and transferred to other neurons.<br>2. Progression of PD may be caused by neuron-to-neuron	[102]

			transmission of α-synuclein fibrils through axonal transport	
	PDMS device with two large open culture chambers connected by a parallel array of microchannels	Mice dopaminergic neurons	1. Visualization of mitochondrial transport in aligned dopaminergic neurons<br>2. Rapid (<1h) and selective decrease of mitochondrial movement upon application of the PD-mimetic toxin MPP	[103]
Multiple sclerosis (MS)	PDMS microfluidic device with two compartments	Neurons and oligodendrocytes differentiated from mouse embryonic stem cells	1. Observation that oligodendrocytes anchor to the bare axons before wrapping them and forming the myelin sheets	[104]
	PDMS microfluidic device with two compartments	Rat primary hippocampal neurons, rat and mouse primary microglia cells	1. Microglia cells contribute to the clearance and phagocytosis of unmyelinated axonal debris	[105]
Amyotrophic lateral sclerosis (ALS)	PDMS microfluidic device with three compartments	Motor neurons (differentiated from mouse embryonic stem cells), $SOD1^{G93A}$-expressing astrocytes and myofibres	1. Motor neurons cocultured with ALS-related *SOD1*^{G93A} astrocytes display loss of axonal projections and reduced myofibre contractions, resembling the early peripheral pathology of ALS seen in humans<br>2. Necrostatin, an ALS drug candidate, was able to reverse the phenotype, improving motor neuron survival and reducing the deterioration of motor innervation	[106]
	Microfluidic device with two chambers	Spinal motor neurons, spinal glial cells and skeletal myocytes	1. Cultured motor neurons recapitulated the in vivo organization<br>2. Motor neuron cell bodies properly grew and spread within a spinal-cord environment, with the support of glia cells, extending towards skeletal muscle to form synapses	[107]
	Microfluidic device with two channels and three wells	*Hb9*:GFP motor neurons (from mouse spinal cord explants) and myotubes	1. The established system is optimized for NMJ cell biology, allowing independent visualization and manipulation of the NMJs at the pre- and postsynaptic cell compartments	[108]
	PDMS microfluidic system with two side perfusion channels and one central channel	Mice primary cortical neurons and astrocytes	1. Cortical neurons in metabolic contact with SOD-mutant astrocytes had a reduction in cell density of about 45% and loss in synapsin protein expression	[109]

			2. Contrastingly, SOD-WT overexpressing astrocytes reduced oxidative stress on the cortical neurons	
	PDMS microfluidic chip with two channels	Human brain microvascular endothelial cells and spinal motor neurons (derived from iPSC)	1. Co-culture resulted in vascular-neural interaction and activation of specific spinal cord developmental genes, enhancing neuronal function and in vivo-like signatures	[110]
	PDMS microfluidic device	Human iPSC-derived motor neurons, skeletal muscle cells and endothelial cells	1. ALS microfluidic model had motor neuron degeneration, increased muscle apoptosis and atrophy and reduced contraction force, compared with control<br>2. Rapamycin and bosotunib co-treatment the disease improved neurotoxicity, motor neuron survival and muscle contraction<br>3. Glutamate excitotoxicity caused motor neuron dysfunction and death, along with neurite regression and muscle atrophy	[111]
	Microfluidic device with two cell culture chambers	Mouse primary cortical neurons	1. β-methylamino-L-alanine (BMAA) caused observing axonal degeneration at sublethal concentrations<br>2. Observation of rapid BMAA transcellular forward spreading between neurons, which could possibly be associated with ALS progression	[112]
Huntington's disease (HD)	PDMS microfluidic device with three compartments	Cortical and striatal neurons (either mHTT-producing, derived from *Hdh*$^{CAG140/+}$ and *Hdh*$^{Q111/+}$ mice embryos, or wild-type, derived from C57/BL6J or CD1 background mice)	1. The genetic status of presynaptic neurons plays a crucial role in HD striatum dysfunction and neurodegeneration: HD cortical neurons expressing mHTT caused functional and signalling impairments in striatal neurons, altering the global integrity of the whole network, while WT cortical neurons were sufficient to rescue and restore the circuit in HD striatum, improving survival signalling	[113]
	PDMS microfluidic system	Human HD patient iPSC	1. Permeability for dextran of several molecular sizes was increased, suggesting a significant disruption of the integrity of the vascular barrier	[114]
